# La Sassa cave: Isotopic evidence for Copper Age and Bronze Age population dynamics in Central Italy

**DOI:** 10.1371/journal.pone.0288637

**Published:** 2023-07-26

**Authors:** Marco Romboni, Ilenia Arienzo, Mauro Antonio Di Vito, Carmine Lubritto, Monica Piochi, Maria Rosa Di Cicco, Olga Rickards, Mario Federico Rolfo, Jan Sevink, Flavio De Angelis, Luca Alessandri

**Affiliations:** 1 Centre of Molecular Anthropology for Ancient DNA Studies, Department of Biology, University of Rome “Tor Vergata”, Rome, Italy; 2 Department of Biology, University of Pisa, Pisa, Italy; 3 National Institute of Geophysics and Volcanology, Vesuvius Observatory, Naples, Italy; 4 Dipartimento di Scienze e Tecnologie Ambientali Biologiche e Farmaceutiche (DISTABiF), Università degli Studi della Campania “Luigi Vanvitelli”, Caserta, Italy; 5 INFN Naples – CHNet, Naples, Italy; 6 Department of History, Culture and Society, University of Rome “Tor Vergata”, Rome, Italy; 7 Institute for Biodiversity and Ecosystem Dynamics (IBED), University of Amsterdam, Amsterdam, The Netherlands; 8 Department of Mental, Physical Health and Preventive Medicine, University of Campania Luigi Vanvitelli, Naples, Italy; 9 Groningen Institute of Archaeology, University of Groningen, Groningen, The Netherlands; 10 Department of Science of Antiquity, University of Rome La Sapienza, Rome, Italy; University of Bern: Universitat Bern, SWITZERLAND

## Abstract

This study focuses on the changes in diet and mobility of people buried in the La Sassa cave (Latium, Central Italy) during the Copper and Bronze Ages to contribute to the understanding of the complex contemporary population dynamics in Central Italy. To that purpose, carbon and nitrogen stable isotope analyses, strontium isotope analyses, and FT-IR evaluations were performed on human and faunal remains from this cave. The stable isotope analyses evidence a slight shift in diet between Copper and Bronze Age individuals, which becomes prominent in an individual, dating from a late phase, when the cave was mainly used as a cultic shelter. This diachronic study documents an increased dietary variability due to the introduction of novel resources in these protohistoric societies, possibly related to the southward spread of northern human groups into Central Italy. This contact between different cultures is also testified by the pottery typology found in the cave. The latter shows an increase in cultural intermingling starting during the beginning of the middle Bronze Age. The local mobility during this phase likely involved multiple communities scattered throughout an area of a few kilometers around the cave, which used the latter as a burial site both in the Copper and Bronze ages.

## Introduction

The complex Copper Age (CA) and Bronze Age (BA) population dynamics in Central Italy have been thoroughly analysed using multifaceted approaches. However, the archaeological, isotopic, and genomic information collected thus far does not fully elucidate the detailed biological and social development of its past populations. In that perspective, it might be beneficial to contribute to unravel the bio-cultural characteristics of people through a diachronic evaluation at the community level in an area already known as a stage for complex cultural and biological dynamics [1–13]. Our study aims to explore these dynamics by studying the differences in diet and mobility patterns of people buried in the La Sassa cave in different periods, accounting also for the typological analyses of contemporary pottery. We performed extensive isotopic and preservation analyses on bones and teeth enamel to characterize the individual mobility and dietary patterns. To establish a local isotopic baseline, we analysed soil and water samples, and faunal remains. We put the results in the context of the recent genomic, isotopic, and typological studies which directly—the first two—and indirectly—the latter–addressed the population dynamics in prehistoric Central Italy [8, 12, 13].

### The La Sassa cave

**Fig 1:**
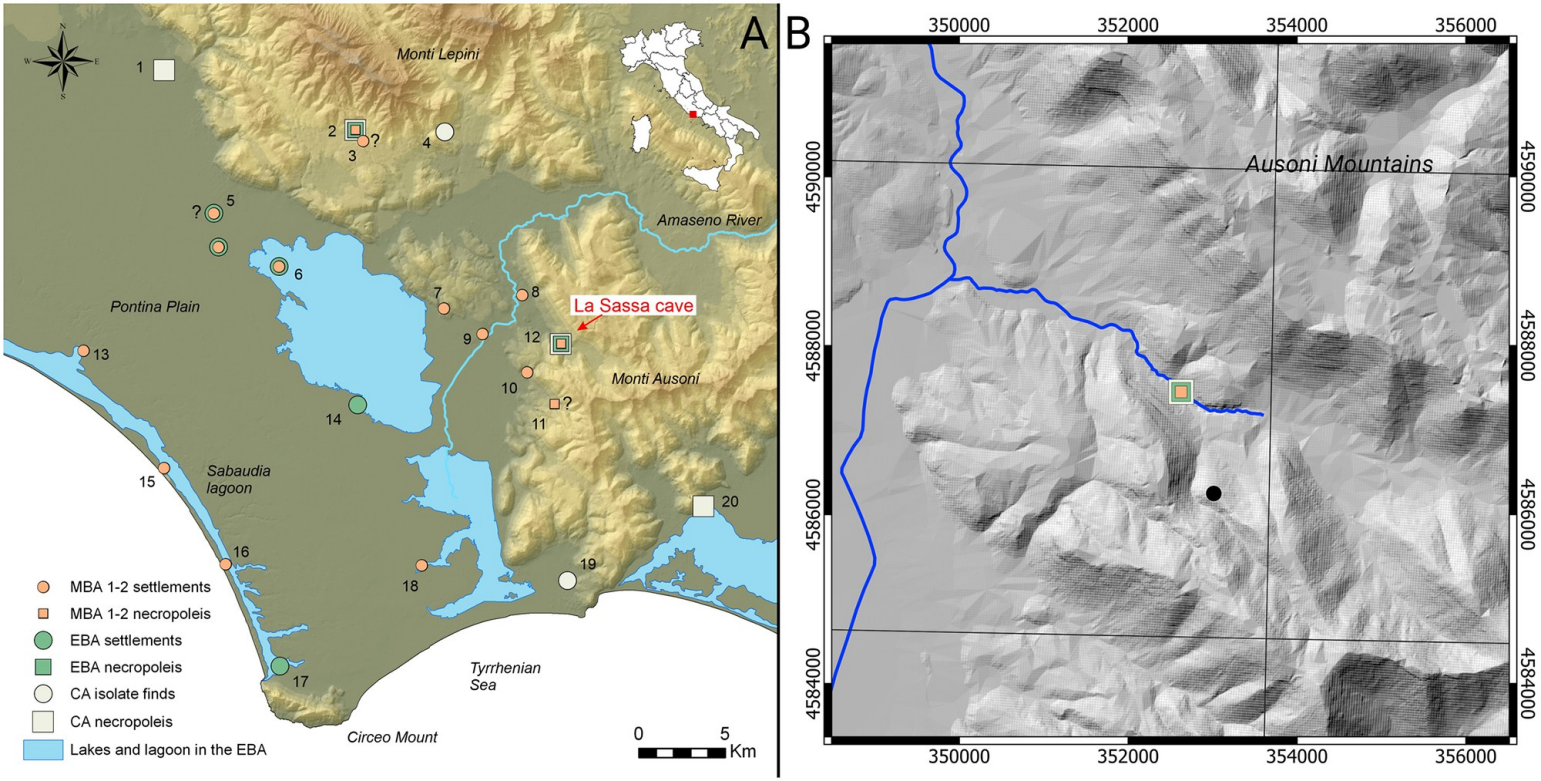
Location of the La Sassa cave. A: La Sassa cave and the CA, EBA, and MBA subphases 1–2 sites. Entrance coordinates: WGS84 / UTM zone 33N (EPSG:32633): 352628E, 4587449N, municipality of Sonnino. 1, Vacchereccia; 2, Grotta Vittorio Vecchi; 3, Longara; 4, Monte Acuto; 5, Tratturo Caniò; 6, Proprietà Ricci; 7, Valle Fredda; 8, Mola dell’Abbadia; 9, Colle Pistasale; 10, Colle Colanero; 11, Pistocchino cave; 12, La Sassa cave; 13, Colle Parito; 14, Mesa; 15, Caprolace; 16, Caterattino; 17, La Casarina; 18, Borgo Ermada; 19, Terracina (in the municipality); 20, Scalelle. The EBA from Tratturo Caniò and the MBA subphases 1–2 from Longara are uncertain. In the Pistocchino cave no archaeological excavation has been performed so far: the interpretation as necropolis is uncertain. B: La Sassa cave on the LiDAR background, grid every 2km, coordinates datum EPSG 32633. Sites from Alessandri [15], Bakels et al. [16], Anastasia [17], Carboni [18] and Tol et al. [19]. Reconstruction of lakes and lagoons from Alessandri [15], van Gorp, Sevink and Van Leusen [20]. Background DEM from TINITALY/01 [21] published with a CC BY 4.0 license, LiDAR from the Italian Ministero dell’Ambiente e della Tutela del Territorio e del Mare, published with a CC BY SA 3.0 license.

The La Sassa cave is in the west of the Monti Ausoni (Fig 1), which form part of the Latium-Abruzzi Meso-Cenozoic carbonate platform of Central Italy. These mountains consist of thickly bedded, relatively homogenous limestones dating back from the Cretaceous to Palaeocene (Carta Geologica d’Italia, sheet 159, Frosinone). The northern extension of the Monti Ausoni borders a series of elongated tectonic basins that form the Amaseno river catchment. The Amaseno first runs through a narrow valley between the Monti Ausoni and aeolian sand-covered limestone hills of Priverno/San Martino and then enters the Agro Pontino graben. There, Holocene deposits prevail, with some outcrops of older lagoonal deposits of Eemian age [14] and alluvial fans and debris slopes descending from the Monti Ausoni (Chapter 1 in S1 File).

**Fig 2:**
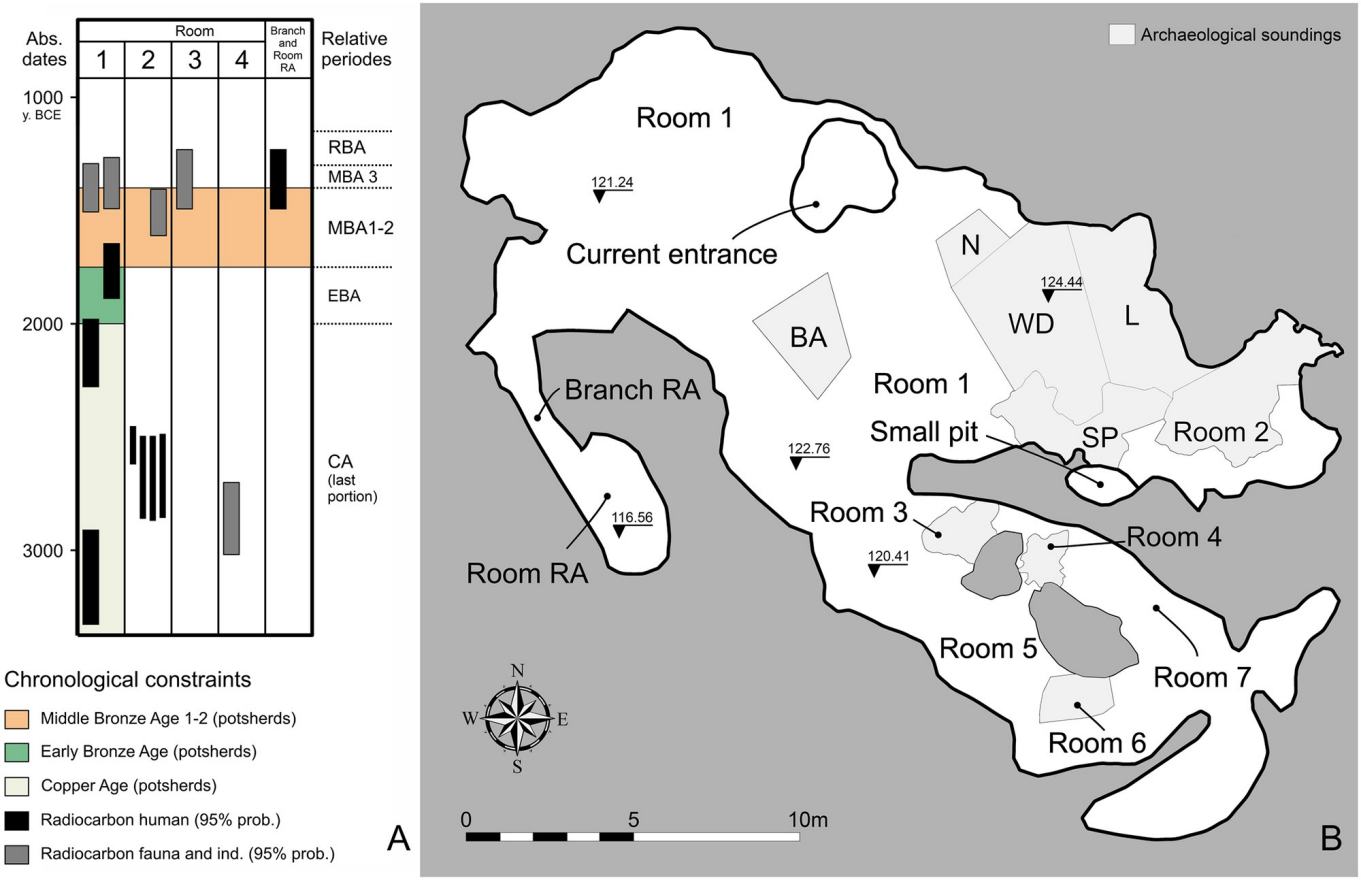
Chronology and location of soundings in the La Sassa cave. A) Chronological constraints for the various rooms of the cave. CA: Copper Age; EBA: Early Bronze Age; MBA: Middle Bronze Age; RBA: Recent Bronze Age. Pleistocene contexts are not indicated. No reliable radiocarbon dates are available for the boundary between MBA subphases 1 and 2 in Central Tyrrhenian Italy. B) Map of the Cave with the name of the different soundings (BA, WD, L, RA, SP, etc.). Outline of the cave from photogrammetric survey (Rooms 1–2 and partly 3) [22, 24], and instrumental survey (Rooms 4–7 and partly 3). Modified after [23], published with a CC BY 4.0 license.

The cave is in the La Sassa valley, which runs from the modern city of Sonnino to the larger Amaseno valley. Since 2015, the La Sassa cave (Sonnino, Latina province, Southern Latium) has been explored in multiple archaeological campaigns [3, 22], yielding a multi-stratified archaeological archive dating from the Late Pleistocene up to the present (Fig 2A). It contained multiple bone assemblages, often with tiny bones originally tied in weak diarthrosis, such as metacarpals and phalanges. For that reason, these assemblages were supposed to be in pristine condition. Human remains were found in three areas (L, WD, and N) in Room 1 (Fig 2B), an area which was probably used later as a cultic shelter, at the beginning of the Middle Bronze Age (MBA). Even though no associated diagnostic potsherds were found, the stratigraphy and the radiocarbon dates for the human bones constrain the age of the strata involved to the Copper Age (CA) and Early Bronze Age (EBA). Human remains, radiocarbon dated to the CA [13, 23] have also been found in Room 2. Moreover, the bones of an infant found in Room RA were radiocarbon dated to sub-phase 2 of the MBA [23].

### Archaeological setting

So far, no systematic study was performed on the possible human activities in the area around the La Sassa cave during the CA and most artifacts were found by chance (Fig 1A). Some isolated items from that period come from the adjacent Monti Lepini and Monti Ausoni [18]. Human burials have been found in two nearby caves: the Scalelle and Vittorio Vecchi caves. The burial goods collected at the Scalelle cave [25] typologically belong to the southern Italian *Gaudo* CA facies [2]; the latter covers in *Latium* a chronological span of about 500 years (3330–2860 BCE) [26]. Hundreds of disarticulated human bones in the Vittorio Vecchi cave have been found with abundant EBA (around 2100–1750 BCE) and MBA (around 1750–1300 BCE) potsherds and a few CA potsherds [4, 5, 15]. Though no radiocarbon dates are available, the funerary use of the cave can be narrowed down to the BA. In the Monti Lepini and Ausoni, EBA artifacts have not been found so far. In the nearby Pontine plain, only a few items have been discovered [7]: an isolated bronze axe in the Sabaudia lagoon [15], and some potsherds below the tephra of the Avellino eruption [27], recently dated at around 1900 BCE [4, 28, 29].

The scarceness of CA and EBA material finds in the mountains might be explained by the sparse and non-systematic research that was executed thus far. In contrast, the paucity of artifacts in the nearby Pontine plain likely reflect a low population density during the CA [15]. Multiple sites arose along the coastline and at the foot of the Monti Ausoni in the next MBA subphases 1 (MBA1, starting at around 1750 BCE) and 2 (MBA2, about 1600–1400 BCE) [15].

At the beginning of the MBA subphases 1–2, the potsherds collected in the Pontine plain show stylistic influences from both the northern *Grotta Nuova* facies [30] and southern *Protoappeninico* facies [31] (both dated to MBA 1–2, around 1750–1400 BCE). Therefore, it was hypothesized that the border between these facies was in the Pontine plain at that time [1, 31]. Furthermore, even though the study of the potsherds collected at La Sassa is still in progress, some preliminary observations show that the typology and style of the CA and the EBA ceramics mostly belong to the southern *Gaudo* and *Palma Campania* cultures, respectively [23, 24]. However, the MBA potsherds from the sounding SP (for details, see Chapter 8 in S1 File) and Room 3 [23] show *Protoappenninico* stylistic traits. A similar situation was observed in the Vittorio Vecchi funerary contexts [5], which is located 17km North-West of the La Sassa cave in the Lepini Mountains and separated from the Ausoni Mountains by the Amaseno valley (Fig 1A). A deposit ranging in age from CA to MBA has been found in the cave, together with hundreds of human bones. The CA and EBA potsherds show parallels only with Central Italy: no potsherds reflect stylistic traits exclusive of the southern facies. In the MBA1, most potsherds show *Grotta Nuova* styles, while few show the *Protoappenninico* style. The chronologies of these stylistic (facies) differences suggest that the Vittorio Vecchi and the La Sassa caves belonged to two different diffusion areas in the CA and EBA, based on the circulation of goods–via ceramics. These observations led us to conclude that the Amaseno Valley likely formed a boundary between the *Grotta Nuova* and *Protoappenninico* facies, which already was hypothesized to have existed in the Pontine Plain [1]. Furthermore, the contacts among people living in the two mountain massifs would have increased in the MBA1, possibly at a late stage, as the only Grotta Nuova potsherd in the La Sassa cave could be dated to the late MBA1.

### Preservation, diet, and mobility

Recently, the evaluation of mobility patterns for prehistoric Italian communities has been boosted through isotope studies on the bones and teeth [8, 9, 32–35]. However, even though they may provide a wealth of information, the success of these biomolecular and biochemical techniques depends on the state of preservation of the samples. Fourier transform infrared (FTIR) spectroscopy is ideally suited to check the preservation of archaeological materials, and thus the reliability of the isotopic analyses on bones [36–40].

FTIR spectroscopy is applied in investigations of human remains in both archaeological and palaeontological contexts [41–48]. FTIR analysis yields information on both the organic and inorganic components through the evaluation of spectra characterized by peaks attributable to specific compounds (i.e., hydroxyapatite and collagen) and their chemical composition [40].

Phosphate and carbonate minerals, as well as collagen, can differ with respect to their structure and abundance, depending on freshness, aging maturation, and post-mortem processes [49, 50]. The presence and relative amount of important functional groups in bones, e.g., HPO_4_^2-^, PO_4_^3-^, CO_3_^2-^, and amide, can be defined by the various FTIR, ATR, DRIFT or pellets modes [45, 50]. Carbonate type (i.e., type A and type B replacing either OH^-^ or PO_4_^3-^, respectively), inorganic calcite, proteins, water, phosphate and fluorapatite, and their relative abundance can be identified [50]. Therefore, the FTIR spectra may provide a simple screening method to assess the state of preservation of proteins and the *in vivo* and post-mortem crystallizations in bones and teeth, as well as structural resemblances, making that analysis mandatory for characterizing the mobility and dietary patterns of past people.

So far, food and drink intake lead to the incorporation of a variety of elements in the body, including carbon (C), nitrogen (N), strontium (Sr), and oxygen (O). Their concentration and isotopic composition vary depending on several interconnected factors, including concentration and isotopic composition in the biosphere, bioavailability, and dietary habits [51]. The carbon and nitrogen isotopic fingerprints obtained from bones and teeth protein fraction are often linked to diet [52]. Still, they might be translated into demic mobility markers and the associated introduction of novel cultural and biological features. Such an approach has already been applied in a multitude of Italian BA funerary contexts [11, 12, 53, 54]. Conversely, Sr isotopes from bones and enamel are typically used as valuable proxies for inferring the mobility of individuals and, provided that an individual’s diet is mainly based on local food and drink, can be used to mark the place of childhood residence [55–57]. The Sr bioavailability and its isotopic composition are also influenced by dietary habits. Accordingly, the Sr fingerprint should be combined with dietary markers [52] such as δ^13^C and δ^15^N for a proper evaluation of the values found. Sr enters the body through the gastrointestinal tract [57] and can substitute calcium in the skeletal carbonate hydroxyapatite, even though Ca is strongly prioritized during incorporation to bone [58].

As Sr isotopes are typically used for inferring individuals’ movements, it seems to be critical to clarify to what extent diet can affect the Sr isotope composition of human skeletal tissue [57]. Terrestrial animal-based food, such as meat, has a relatively low Sr/Ca ratio compared to plants and fish (both marine and freshwater), contributing less to the bone—or teeth enamel—Sr fingerprint [57]. Thus, individuals relying on terrestrial animal-based food will have their Sr signature less influenced by the food Sr signatures, making the mobility identification less prone to misleading interpretations [57].

### Bioarcheological evidence of regional mobility in the Italian context

A range of biochemical markers has been used to infer individual mobility. Still, studies on the mobility in Central Italian CA and BA communities based on isotopic analyses are currently scant, and any reconstruction remains rather tentative. De Angelis and colleagues [8] used the oxygen isotopes to identify a sedentary lifestyle rather than extensive mobility in Central Italian CA burials. Their results were consistent with those obtained in strontium (Sr) isotopic studies for some Southern Italian sites [33]. Conversely, the isotopic data for Northern Italian communities indicated that mobility strongly increased during the BA [34].

Concerning the diet, Central Italian CA and BA communities had a terrestrial diet, mainly based on C3 plants typical of the ’Neolithic package’ (wheat and barley) with a moderate animal protein intake. At the same time, alternative crops (C4 plants, e.g., *Setaria italica* foxtail millet and *Panicum miliaceum* broomcorn millet, hereafter referred to as millets) were progressively introduced into the Italian peninsula in the MBA, though their consumption may have been limited to occasional periods or particular sites [9–12, 35].

C4 plants are characterized by increased adaptability to adverse environmental conditions and frequent harvesting, making them suitable for challenging environments. These species are thought to have been introduced into Europe from the Eurasian region during the Neolithic and arrived in the Italian peninsula in the BA [10, 12, 32, 59]. So far, the spread of C4 plant consumption seemed to be restricted to Northern and Central Italy in the MBA. The southernmost fringe of their consumption was detected in the MBA Misa cave (Ischia di Castro, VT) and, possibly, at the MBA 1–2 Felcetone cave (Ischia di Castro, VT) in the Latium/Tuscany border area [11]. Furthermore, some consumption of C4 plants was detected through the identification of starch grains in the dental calculus on teeth found in the Scoglietto cave (Grosseto, GR) [60], and recently in a diachronic study of teeth from the Continenza cave site in the Abruzzi (Trasacco, AQ) [61]. Though it is not fully understood whether these C4 plants were cultivated and consumed by people, gathered as wild plants, or served as animal fodder as suggested for continental communities [62, 63], it is worth noting that the diet of people living in the Italian peninsula during the BA seemed to have primarily consisted of C3 plant and a moderate amount of animal protein rather than C4 plant resources [12, 35].

Other than the isotopic evidence related to the spread of C4 plant exploitation, results from recent ancient human genomics studies support the southward flow of people carrying Steppe-related ancestries into Central Italy during the BA [13]. Such lineages were found in MBA people buried in the Grotta Regina Margherita, which carried different genetic signatures with respect to CA people from the La Sassa cave [6, 13].

## Material and methods

### The archaeological excavation

In 2015, the La Sassa cave was mapped, and the surface artifacts collected. From 2016 to 2019, four excavation campaigns took place led by the Groningen Institute of Archaeology (Groningen University, Netherlands), in collaboration with the University of Rome Tor Vergata (Rome, Italy). Excavation and study permits have been received yearly from the Soprintendenza Archeologia, Belle Arti e Paesaggio per le Province di Frosinone, Latina e Rieti (2016: n. 4888; 2017: n. 9559; 2018: n. 13261; 2019: n. 16086). The cave area has been subdivided into progressively numbered rooms. Several soundings have been excavated in different parts of the cave. When the sounding area equals the room, the former has been labelled the same (Sounding Room 3); otherwise, an alphabetic code has been assigned to each sounding (sounding L, WD, SP, BA, all in Room 1) (Fig 2B). The human and faunal remains have been individually collected and a progressive number with a prefix (LS) was assigned to each of them (LS1, LS2 etc). They have been recorded in a local coordinate system and afterwards were reprojected into the WGS84, UTM zone 33N (EPSG: 32633). The relative chronology is based on typo-chronological parallels and stratigraphic considerations, the absolute chronology is based on radiocarbon dates [13, 23].

### The selection of biological remains

The analysed skeletal series consists of over 800 pieces of human bones or teeth, mainly recovered in Room 1 and Room 2 in a chaotic bone assemblage, probably due to taphonomic processes, and preventing the identification of individual burials. However, a comprehensive classification of each informative remain was performed according to bone or tooth, side, and, whenever possible, osteological markers that allow for estimations of sex and age at death.

The age of death was assessed by the wear of the dental occlusal surface, the level of formation of the dental roots, and the synostosis degree of the tubular bones [64, 65]. Sex identification was performed on dimorphic skeletal districts, mainly focusing on hipbones and skull traits [66, 67].

The mandible was the most recovered anatomical district that allows for identifying at least 17 individuals (Minimum Number of Individuals; MNI = 17) buried in Room1 and Room2. However, merging the information about sex, age at death, osteological characterization, and osteometric analysis of the whole osteological material, we suggest a reliable estimate of at least 27 individuals.

We selected fifteen mandibles, a maxillary bone, six femurs, and 25 teeth for tracing the 87Sr/86Sr ratio among the individuals. The selection was driven by macroscopic preservation (S3 Table in S1 File).

Due to the impossibility of identifying whether some anatomically disarticulated bones pertain to a single individual, we selected 46 bones representative of the whole set of bones identified to explore the carbon and nitrogen stable isotopes ratios, even though multiple districts could be redundant [68, 69]. However, we limited our interpretation on the data gained through the evaluation of the MNI and the adjunct individual from RA.

We isotopically characterized seven faunal remains (Table 3) to account for the ecological background.

To set the Sr isotopic baseline within and around La Sassa cave, six groundwater and seven soil samples were analysed for their Sr ratio (S2 Table in S1 File). We decided to collect soil and water samples in a range of 5 km around the cave to characterize the ‘local baseline’, as the BA communities were supposed to exploit a territory with an average walking time consistent with this distance [15], and analysed two more distant soil samples (Fig 6, Soil6 and Soil7) to get a more comprehensive view.

### Sample preparation and analytical conditions for FTIR analyses

FTIR was used to investigate biominerals and collagen preservation [45] in selected samples based on their Sr-isotope determinations. Carbonate type A, carbonate type B, inorganic calcite, proteins, water, phosphate, fluorapatite, and their relative abundance were determined by means of the diffuse reflectance infrared spectroscopy (DRIFT-FTIR) at the Istituto Nazionale di Geofisica e Vulcanologia, Sezione di Napoli Osservatorio Vesuviano (INGV, OV) (Naples, Italy), where a Nicolet 670 NexusTM equipped with a DRIFT accessory (ThermoFisher Scientific S.p.a) was available. The configuration included a heated ceramic (Globar) source, 670 Laser unit, KBr beam splitter and an MCT detector. The reference 3.0 mil Polystyrene ASS0100421 by Nicolet Instrument Corp. was preliminary used as process control. Bone powders were mixed with KBr in an agate mortar in a ratio of 1:10.

Spectra were recorded on the mixture following the background attainment on the KBr alone at 100 scans and a resolution of 8 in the 5000–400 cm^−1^ range. For data acquisition and interpretation, we used the OMNIC Data Collector 5.2© software. Some samples have been duplicated or triplicated to assess analysis reproducibility. The errors in absorbance are on the order of 0.003; the various amine bands in the 1600–1450 cm^-1^ region are weak and caused largest uncertainty in absorption evaluation. In applying DRIFT-FTIR technique, we careful considered the literature suggestions about possible uncertainties compared to ATR and pellets [45]. The indices in Table 1 were calculated by using the line between 1900–900 and 800–500 (750–400) cm^-1^ [41, 43, 45]. Based on previous studies ADD REF, the indices in Table 1 were calculated by using the line between 1900–900 and 800–500 (750–400) cm^-1^. The relative amount of carbonate vs. phosphate (CC/PP) was calculated from the ratio between the sum of absorbances at 1460 and 1425 cm^-1^ and the sum of absorbances at 605 and 568 cm^-1^ by using the baseline defined between 500 and 2000 cm^-1^ [42]: (A_1460_+A_1425_)/(A_605_+A_568_). Errors in the index have been determined considering differences among samples analysed in duplicate or triplicate.

**Table 1:** Infrared indexes with their significance and formula.

BPI	Relative abundance of type B carbonate to phosphate: absorbance at 1415 cm^-1^ divided by the absorbance at 605 cm^-1^(**A**_**1415**_**/A**_**605**_)
API	Relative abundance of type A carbonate to phosphate: absorbance at 1540 cm^-1^ divided by the absorbance at 605 cm^-1^ (**A**_**1540**_**/A**_**605**_)
BAI	Relative amount of B to A carbonate: absorbance at 1415 cm^-1^ divided by the absorbance at 1540 cm^-1^ (**A**_**1415**_**/A**_**1540**_)
PCI	Crystallinity: sum of the absorbances of the phosphate bands at 565 cm^-1^ and 605 cm^-1^ divided by the absorbance at 590 cm^-1^ (A_565_+A_605_)/(A_590_);
C/P	Relative amount of carbonate vs. phosphate: absorbance at 1415 cm^-1^ divided by absorbance at 1035 cm^-1^ (**A**_**1415**_**/A**_**1035**_)
Am/P	Collagen content of bones: absorbance of organic component at 1640 cm^− 1^ divided by the absorbance of ν_1_PO_4_ at 1035 cm^-1^ vibrational mode (A_1640_/A_1035_)

### Sample preparation and analytical conditions for Sr isotope ratio determination

Tooth and bone samples from La Sassa, soil and water samples from the surroundings were prepared in the Clean Chemistry Laboratory of Istituto Nazionale di Geofisica e Vulcanologia, Sezione di Napoli Osservatorio Vesuviano (INGV, OV). Whole teeth were washed 2–3 times with Milli Q^®^ H_2_O:H_2_O_2_ = 3:1 in an ultrasonic bath in to remove organic material. Following this phase, a small piece was cut from each tooth with a dentist milling machine and Milli Q^®^ H_2_O as a lubricant and washed again in an ultrasonic bath with a mixture of Milli Q^®^ H_2_O:H_2_O_2_ = 3:1. Pieces of jawbones were washed in an ultrasonic bath 2–3 times with Milli Q^®^ H_2_O at first, and then several times with a mixture of Milli Q^®^ H_2_O:H_2_O_2_ = 3:1, to remove organic material. Once cleaned, samples were dissolved by using ultrapure HNO_3_ 65% in closed Savillex^®^ vials. Following dissolution, the acid solution was evaporated till dry on a hot plate. The obtained solid fractions were dissolved in ultrapure 2.5N HCl and centrifuged for 10 minutes at 5000 rpm. Solutions were then loaded on quartz columns for the chemical separation of Sr by standard Sr isotope geochemistry procedures [69–71].

About 5 grams of soil samples taken within a radius of 5 km from the cave were left spinning in 100 ml of 1M ammonium acetate for 12 hours. Solutions were then filtered by using 0.45 microns filters, dried on a hot plate, and dissolved in ultrapure 6N HCl at first, and 2.5N HCl at last. From the obtained, centrifuged acid solution, a 0.5 ml aliquot has been loaded on quartz columns for the chromatographic separation of Sr.

Aliquots of groundwater, originating from the Apennine chain, and streaming in vicinity of the La Sassa cave, have been analysed with the aim of characterizing the local aquifers. Samples were evaporated till dry, and the residues dissolved in ultrapure 2.5 N HCl. Before chemical separation of Sr, solutions were centrifuged for 10 minutes at 5000 rpm.

The Sr fractions dissolved in diluted HNO_3_ have been loaded on previously degassed zone refined Rhenium filaments, to carry out the measurement of ^87^Sr/^86^Sr isotope ratios by thermal ionization mass spectrometry (TIMS) techniques at the Radiogenic Isotope Laboratory of the INGV, OV. Determinations were performed with a ThermoFinnigan Triton TI multicollector mass spectrometer running in static mode. Measured ^87^Sr/^86^Sr ratios were normalized for within-run isotopic fractionation to ^86^Sr/^88^Sr = 0.1194. For each single measurement, the average 2σmean, i.e., the standard error with N = 180, was ±0.000009. The mean measured value of ^87^Sr/^86^Sr for the NIST-SRM 987 international standard was 0.71023±0.00002 (2σ, N = 171); external reproducibility (2σ) during the period of measurements was calculated according to [72]. The measured Sr isotope ratios were normalized to the recommended value of NIST-SRM 987 (^87^Sr/^86^Sr = 0.71025).

### Sample preparation and analytical conditions for carbon and nitrogen isotope analyses

The collagen extraction from bones was individually performed following Longin’s protocol modified by Brown and colleagues [73, 74], which was also simultaneously applied to a modern bovine sample as a reference. To obtain an acceptable yield of collagen, the extraction was performed on about 500 mg of bone powder collected by drilling. A concentration step was also carried out for all the samples to enhance the collagen yield through 30 kDa Amicon ^®^ Ultra-4 Centrifugal Filter Units with Ultracel^®^ membranes. Each sample of collagen weighed 0.8–1.2 mg and was analysed using an elemental analyzer isotope ratio mass spectrometer at the iCONa (isotope Carbon, Oxygen and Nitrogen Analysis) Laboratory of the University of Campania. Carbon (δ^13^C) and nitrogen (δ^15^N) stable isotope ratios were measured in a single run on a Delta V Advantage isotope ratio mass spectrometer coupled to a Flash 1112 Elemental Analyser via a Conflow III interface (Thermo Scientific Milan, Italy). Results were expressed in δ notation [75] and reported in permill units. The measurements of δ^13^C were calibrated to the international standard VPDB with the standard reference materials IAEA-CH3, IAEA-CH6 and stable isotope ratio facility for environmental research at the University of Utah (SIRFER) yeast; δ^15^N measurements were calibrated to the international standard AIR with the standard reference materials USGS-34, IAEA-N-2 and SIRFER yeast. Typical analytical precision, evaluated from a repeated measurement of an internal standard, was 0.1‰ for δ^13^C and 0.2‰ for δ^15^N. The reliability of the procedure and the exclusion of exogenous contamination were accounted for through a comparison against established criteria to ascertain the percentages of carbon and nitrogen, atomic C/N ratios, and collagen yields [76–78].

The carbon and nitrogen contents and C/N ratio are listed in Table 2. To assess the preservation state of the extracted collagen, we considered carbon and nitrogen contents between 15–51% and 5–18%, respectively [76], and C/N ratios within the range 2.9 to 3.6 [77]. The extraction yield was not used as a criterion [76] as the ultrafiltration technique was applied, and only samples with a yield of 0% were ruled out.

**Table 2:** Stable carbon and nitrogen isotope ratios in human bone collagen from the La Sassa cave.

Samples	US	Bone	Chronology	δ^13^C (‰) vs. VPDB	δ^15^N (‰) vs. Air	%N	%C	C/N	Collagen yield
LS120	19	Mandible	CA	-19.1	9.9	15.0	43.9	3.4	14.1
LS150	19	Mandible	CA	-19.7	9.8	13.9	43.7	3.6	5.5
LS212	19	Mandible	CA	-19.3	9.8	16.4	48.3	3.4	2.1
LS1956	19	Mandible	CA	-19.6	9.4	17.2	54.0	3.6	9.3
LS565	55	Mandible	CA	-20.7	8.4	13.9	41.7	3.5	10.8
LS578	55	Mandible	CA	-19.8	8.5	17.6	53.9	3.6	3.9
LS 865	78	Mandible	EBA	-20.5	8.7	13.3	41.1	3.6	7.6
LS882	78	Mandible	EBA	-20.1	8.7	16.1	46.7	3.4	6.5
LS916	78	Mandible	EBA	-20.3	7.4	12.3	38.4	3.6	2.2
LS1978	78	Mandible	EBA	-20.4	7.4	13.8	42.8	3.6	4.8
LS2161	78	Mandible	EBA	-20.7	8.2	12.2	37.1	3.5	3.1
LS2177	78	Mandible	EBA	-20.7	8.3	14.8	43.3	3.4	11.7
LS2178	78	Mandible	EBA	-20.7	8.3	12.6	38.0	3.5	9.8
LS2203	78	Mandible	EBA	-20.5	9.1	14.3	44.9	3.6	6.7
LS2208	78	Mandible	EBA	-20.5	9.1	13.1	41.3	3.5	5.3
LS2403	97	Mandible	CA	-20.5	8.6	15.3	47.6	3.6	3.3
LS 4852	97	Mandible	CA	-20.2	9.1	15.4	45.1	3.4	4.5
LS2176	RA	Femur L	MBA2	-14.3	10.6	12.3	35.4	3.3	9.3

Descriptive statistics and comparison tests were performed by R v.3.6.1 [79].

The linear mixing model proposed by Fraser et al. [80] and recently developed by Fontanals-Coll et al. [81] was implemented to detect the role of humans as compared to the available ecosystem resources. A theoretical terrestrial endpoint at -20.5‰ ± 0.9 was estimated (-21.5‰ adjusted to +1‰ for fractionation processes due to prey-predator relationship) from the mean δ^13^C value of terrestrial fauna. In the same way, a theoretical marine endpoint of about -12.6‰ ± 3.0 [82, 83] was estimated. The theoretical thresholds for δ^15^N accounting for animal protein consumption were calculated adjusting for 4‰ the average faunal δ^15^N value (5.7‰± 1.5). All the above procedures resulted in a range of 8.2–11.2‰ for terrestrial faunal protein consumption in humans. Similarly, a theoretical marine threshold of about 10.5–14.5‰ was established. The Bayesian reconstruction by FRUITS v.3 [84] was also employed to support our interpretation, using the details in Cortese et al. 2022 [85].

## Results

### FTIR analyses

FTIR analyses were performed on 14 bones and, as an exploratory sample, one tooth (Chapter 3 in S1 File) to test the reliability of the Sr-isotope data obtained. All FTIR spectra are similar (S3 Fig in S1 File), showing a hump in the OH- stretching region overlapped by signals at ca. 2960 and 2880 cm^−1^ from ν(CH-)lipids, a strong peak at ca. 1990–2000 cm^−1^ from cyanamide, and several absorption bands between ca. 1794 cm^−1^ and 400 cm^−1^ linked to the most important functional groups in bone, which are HPO_4_^2-^, PO_4_^3-^, CO_3_^2-^ and amide [41–47]. The spectra also show distinct absorption peaks for ν1 (PO_4_^3-^, the absorption peak at ca. 1045 cm^-1^) and fluorapatite (at ca. 1090 cm^-1^), pointing at an expected (but not complete) deproteinization as compared to fresh (modern) bones [45, 86] (for details, see Chapter 3 in S1 File).

For all samples, the C/P index [45] is above 0.34 and significantly higher than 0.15, which is considered as a threshold value for ‘altered bones’. In addition, the order degree of the phosphate minerals (CPI) varies between 2.7 and 3.4. Although these values might be slightly overestimated by DRIFT, they are generally lower than 3.4, which is the accepted boundary value for the distinction between modern and archaeological bones. Even though there are relatively de-proteinized (i.e., LS2208, LS151) and/or de-phosphatized samples (i.e., LS120, and LS1978) (S4 Fig in S1 File) [41], the FTIR suggests that the Sr isotopic data reflect the original isotopic composition of the bones and that the taphonomic processes have not altered the chemical composition of infrared-investigated samples.

### Strontium isotopes

Though Sr isoscapes are available for Italy [87] at regional scale, these provide inadequate information at more local scale, for which reason we performed an accurate sampling and analysis program of local soil and water samples with the aim of establishing an appropriate local baseline. We determined the Sr isotopic composition of 5 spring-waters and 7 soil samples collected within a radius of ca. 5km around the cave (S2 Table in S1 File). Our environmental data (soil and water samples) are consistent with the isoscapes obtained by Lugli et. al. [87]. Soil samples have Sr isotope ratios that ranged from 0.70983 to 0.70799 (sampled within the cave). The Sr isotope ratio of the cave sediment is the lowest. Moreover, three soils are isotopically similar to limestone from the Monti Lepini (0.7088) [88] and Miocene limestone sampled on the Monti Aurunci (0.7085) [89]. The remaining soil samples are enriched in radiogenic Sr, and ratios fall within the range for volcanic products from the Middle Latin Valley (Volsci Volcanic Field) [88]. Tooth enamels of domestic fauna show a wide range in ^87^Sr/^86^Sr ratio (0.70806–0.70915) (S3 Table in S1 File), with the highest value for the LS419 *Equus sp*. enamel. Local microfauna tooth enamel (*Microtus sp*.) displays the lowest Sr isotopic ratios (0.70803; S3 Table in S1 File).

Sr isotope ratios for human teeth enamel and bones range from 0.70822 to 0.70955 and 0.70810 to 0.70894, respectively (S3 Table in S1 File). ^87^Sr/^86^Sr ratios for bones from LS150, LS212, LS882 and LS2176 resemble that of the cave soil, while those for LS2177, LS2161, LS865 and LS2208 are higher (S3 Table in S1 File). Moreover, some individuals have teeth enamel and bones with similar ^87^Sr/^86S^ratio (LS896, LS2203, LS2208, LS2176, and LS2177), while in others (LS150, LS1956, and LS882) these ratios differ (S2 Fig in S1 File). An inverse correlation exists between ^87^Sr/^86^Sr and FTIR data (S4 Fig in S1 File).

### Carbon and nitrogen isotopes

Though we performed isotopic analyses on an extended sample of 46 human bone specimens (S4 Table in S1 File), in the Table 2 we report the isotope analysis data for the samples accounting for the NMI (17 individuals, according to the mandible presence) and the sole individual in the RA, and thus an overall set of 18 individuals. They all fit the quality criteria for collagen preservation [76–78] (Table 2).

The isotope ratios of faunal remains have been established to characterize the putative prey for people buried in La Sassa cave, even though most of the faunal remains date to the BA (Table 3).

**Table 3:** Stable carbon and nitrogen isotope ratios in animal bone collagen from the La Sassa cave.

Samples	Period	US	Species	%N	%C	δ^13^C (‰) vs. VPDB	δ^15^N (‰) vs. Air	C/N	Collagen yield
LS481	N/A	59	*Bos taurus*	16	45.5	-20.5	9	3.3	7.4
LS514	N/A	59	*Sus domesticus*	15.3	44.8	-20.9	5.2	3.4	11.5
LS793	BA	49	*Bos taurus*	14.7	41.8	-22.5	5.5	3.3	5.8
LS595	CA	55	*Ovis vel capra*	12.9	35.6	-21.7	4.7	3.2	3.2
LS535	BA	49	*Cervus elaphus*	13.6	38.7	-22.4	5.3	3.3	8.6
LS794	BA	49	*Bos taurus*	14.3	39.4	-21.8	5.5	3.2	2.5
LS517	N/A	59	*Sus domesticus*	12.4	35.3	-21.2	4.9	3.3	7.2

The faunal δ^13^C and δ^15^N values range between -22.5‰ to -20.1‰ and 4.7‰ to 9‰, respectively. Overall, these herbivore isotopic data is consistent with a terrestrial C3-based feed intake characteristic of the European ecosystems [90].

**Fig 3:**
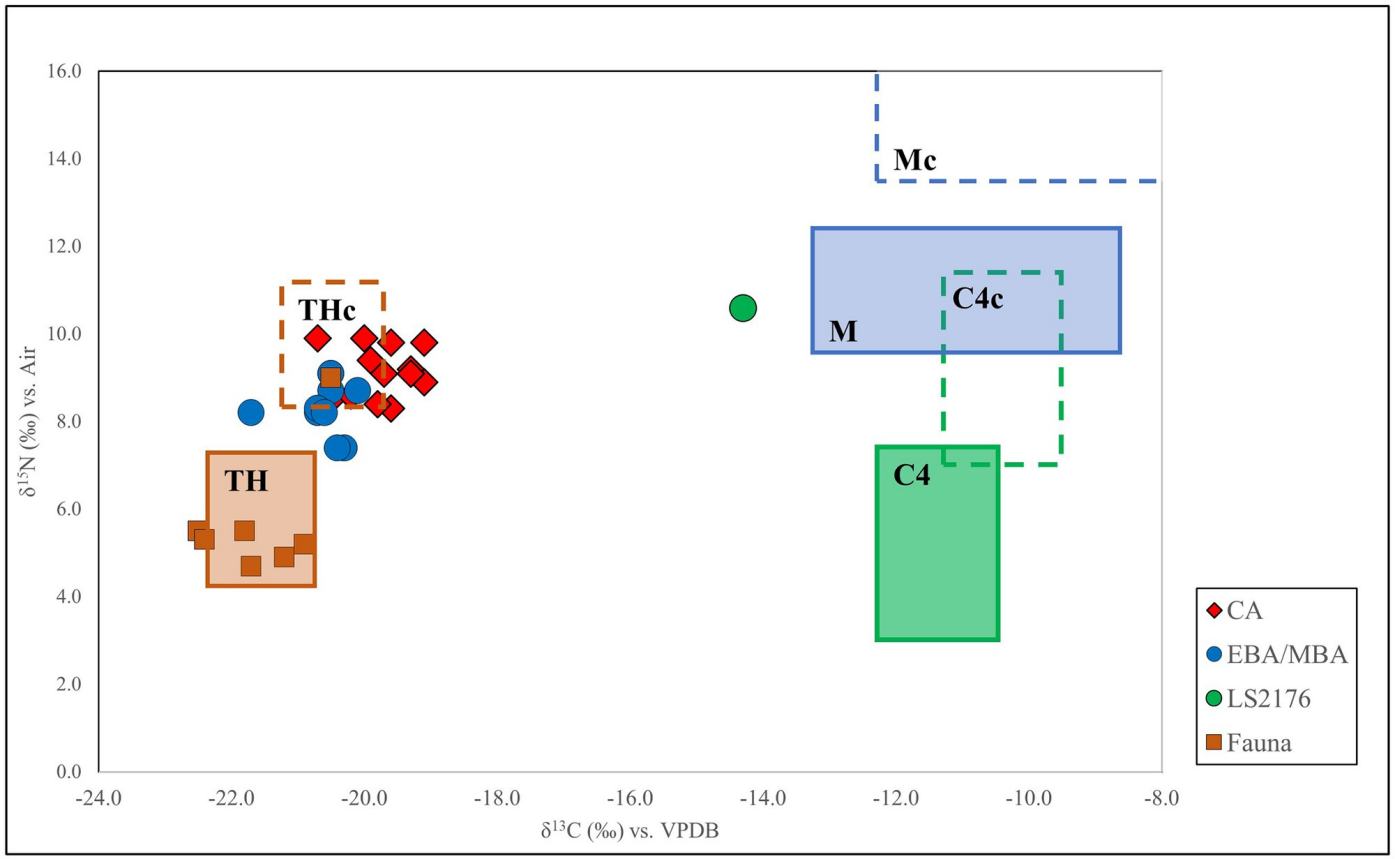
Plot of δ^13^C and δ^15^N isotope ratios. The faunal data cluster according to their habitat and position in the trophic chain (rectangular boxes). The faunal rectangular boxes have been reconstructed using the linear model proposed by Fraser et t al. [80]. The rectangular dotted line boxes are estimated from the faunal rectangular boxes, shifted by 1‰ on the δ^13^C axis and 4‰ on the δ^15^N axis to account for the trophic shift in isotope values from food source to consumer. TH, M and C4: terrestrial herbivores, marine fauna, and C4 plants, respectively. THc, Mc and C4c: terrestrial herbivore consumers, marine resource consumers, and C4 plants resources consumers, respectively.

δ^13^C values in the 18 human remains range from -14.3 to -20.7 ‰, while the δ^15^N values range from 7.4 to 10.6 ‰. Noteworthy, the N and C isotope values found for LS2176 (δ^15^N = 10.6 ‰ and δ^13^C = -14.3 ‰) clearly differ from those for the other samples (Fig 3).

The faunal average values (δ^13^C = -21.5 ‰ ± 0.9 s.d. and δ^15^N = 5.7‰ ± 1.5 s.d.) were used to predict the local baseline for the trophic food chain. These values are compared to those of humans, as reconstructed by the linear model proposed by Fraser et al. [80] and Fontanals-Coll et al. [81] (Fig 3). All CA and EBA/MBA individuals from Room 1 and Room 2 fall in the proximity of the area encompassing the terrestrial herbivore consumers (THc). Conversely, LS2176 from RA is at odds, being positioned far away from the THc box and suggesting a different diet typology.

## Discussion

Due to its location and the chronology of its exploitation, the La Sassa cave offers outstanding possibilities for analysing the complex demographic and economic dynamics of the CA/BA boundary in Central Italy. Evidently, such analysis is complicated by the notoriously problematic dating of chaotic bone assemblages. The radiocarbon dating performed on multiple finds allowed us to date people recovered in Rooms 1 and 2 to a roughly continuous phase starting in the CA and extending into the EBA. The remains of a toddler (LS2176), found in Room RA and unrelated to the bone assemblages in Rooms 1 and 2, followed the latter and probably represent one of the last BA individuals buried inside the cave.

We performed isotopic analyses on an extended sample of 46 human bone specimens (S4 Table in S1 File), but we move through the discussion considering only 18 samples (MNI and the individual from the RA) to avoid potentially over-representation of people buried in the cave.

The values for C and N isotopes obtained for bones recovered in Rooms 1 and 2 are consistent with a diet mainly based on C3 plants-derived ecosystem (Fig 3). The nitrogen signatures are consistent with a moderate protein intake, which points to consumption of animal food. However, the isotopic signatures of the La Sassa CA people were significantly different from that of the BA people (Mann-Whitney δ^13^C U = 13.5; p = 0.03; δ^15^N U = 13; p = 0.04), suggesting a slight change in diet over time. Remarkably, the difference between CA and BA is also recognizable in the extended sample listed in S4 Table in S1 File.

The difference in δ^15^N values may be due to different isotopic ratios of the putative CA prey instead of different dietary habits. To account for that potential explanation and overcome the lack of local CA remains, we collected all the published CA and BA faunal isotopic data from Latium [6, 10, 91, 92] and other Italian regions (S5 Table in S1 File).

These data did not show significant differences between CA and BA (Mann-Whitney δ^13^C U = 325.5; p = 0.05 Mann-Whitney δ^15^N U = 451; p = 0.88). Accordingly, we considered the shift towards lower δ^15^N and slightly more negative δ^13^C values in the BA individuals as consistent with a drop in protein consumption and a diet mostly based on farming in the BA.

To explore the dietary asset of people buried in the La Sassa cave, we compared the data obtained for people from the CA layers with those from roughly coeval Central Italian contexts (S6 Table in S1 File).

First, we used the faunal values collected throughout the peninsula to address putative environmental differences, potentially misleading the evaluation (S5 Table in S1 File). While δ^13^C values are consistent for the sites, a significant variation in δ^15^N was found for the Campanian sites [91]. Because of these odd results and the impossibility of gaining information about the ontogenetic stage of the samples from that area, we excluded them from further evaluations.

Overall, all human individuals showed relatively low values of δ^13^C, suggesting a consistent C3 plant-based diet regime [9] (S6 Table in S1 File) if we did not count communities represented by too tiny sample size, such as Podere Cucule (n = 3). Conversely, δ^15^N values are heterogeneous (S6 Table in S1 File), supporting a local exploitation strategy with different amounts of protein consumption.

A deeper knowledge concerning the Italian BA communities has been recently acquired through isotopic studies highlighting socio-economic transitions and new dietary habits based on the introduction of novel resources such as C4 plants [12, 35, 54, 59] (S7 Table in S1 File).

**Fig 4:**
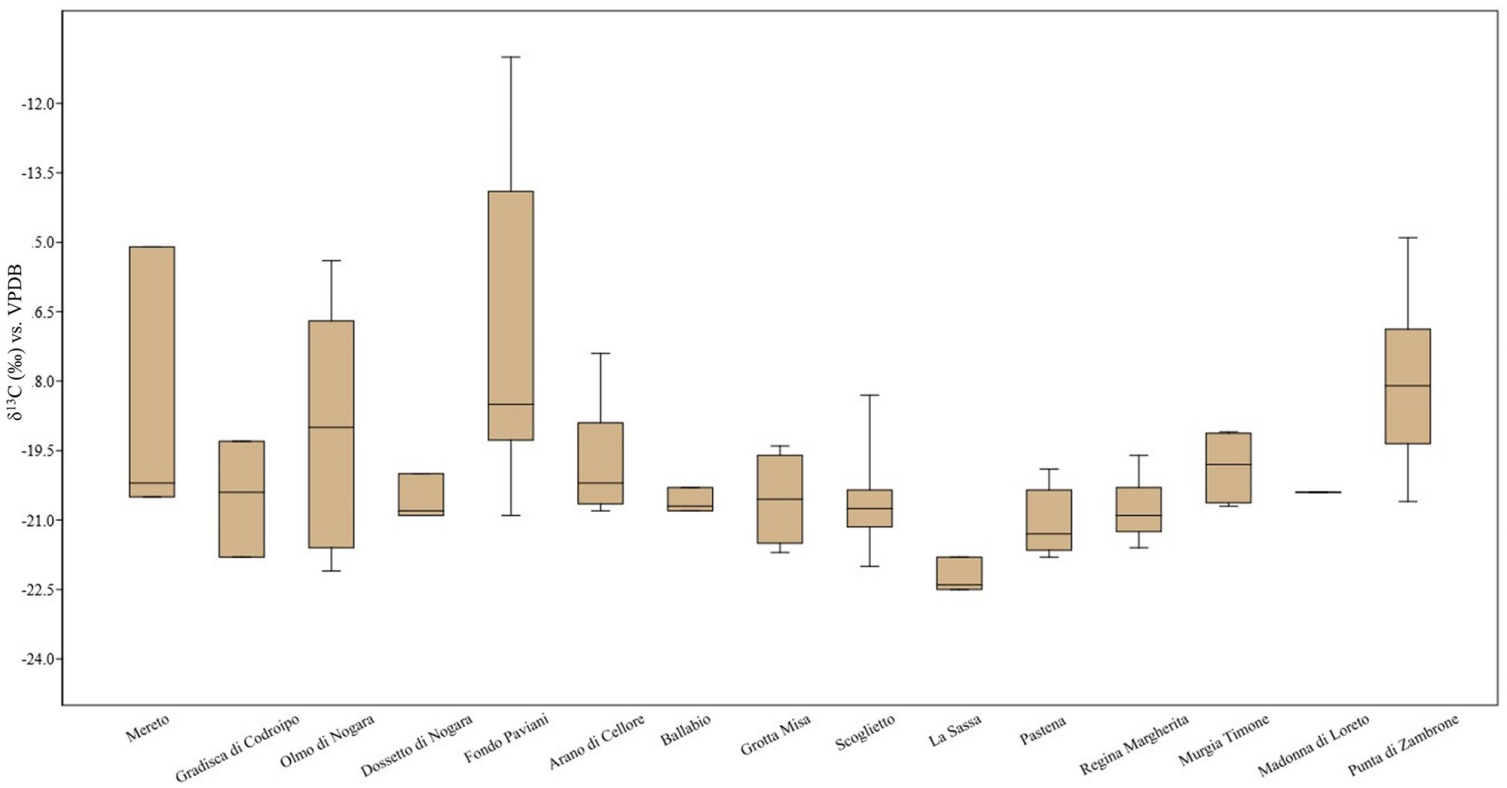
Boxplot of δ13C in the Italian Bronze Age herbivore. Mereto(sample size, N = 3) [32, 59], Gradisca di Codroipo(N = 3) [59], Olmo di Nogara(N = 7) [59], Dossetto di Nogara(N = 3) [59], Fondo Paviani(N = 18) [59], Arano di Cellore(N = 13) [53], Ballabio(N = 3) [54], Grotta Misa(N = 4) [11], Scoglietto(N = 10) [11], La Sassa (this study; N = 3), Pastena(N = 5) [10], Regina Margherita(N = 9) [6], Murgia Timone(N = 4) [35], Madonna di Loreto(N = 2) [32], Punta di Zambrone(N = 10) [93].

So far, data from faunal remains from Latium showed lower δ^13^C values than animals from sites where a consistent consumption of C4 was supposed to occur [59, 93]. This evidence allows us to exclude those resources from the staple dietary setting of Latium herbivores in the BA (Fig 4). However, the results cannot exclude a C4 intake for BA human individuals, even though there is no direct evidence for a C4 plant introduction in the diet of Pontine plain communities in the initial phases of the BA.

Surprisingly, the data for LS2176 from the La Sassa cave suggest a different situation in the late phase of the frequentation of the cave, i.e., during an advanced phase of the BA.

LS2176 was an isolated infant (ca. 1–2 years) in the South-Western fringe of the cave. The radiometric dating (LTL20395A, 1519–1306 calBC, details in S8 Table in S1 File) sets this individual between the MBA sub-phase 2 and the Recent Bronze Age (RBA). Since cremation was the exclusive funeral ritual during the RBA [94], it likely dates from the MBA sub-phase 2 but cannot be later than the MBA sub-phase 3. The δ^13^C value is surprising (-14.3 ‰) and points to a different diet. This toddler is not related to the other BA people discovered in the cave because of its different chronology and location. Unfortunately, the area where the infant was found cannot be further explored due to a massive landslide [23]. Thus, to date, it represents the only individual dated to the MBA sub-phase 2 (or 3) in the La Sassa cave.

LS2176 was probably still breastfed due to the estimation of the age of death, for which reason its odd δ^13^C and δ^15^N values must also relate to the mother’s diet. However, since breastfeeding/weaning progress is associated with exponential decreases of δ^15^N and δ^13^C values with age and with the loss of one trophic level (ca. 4‰ and ca. 1‰, respectively) [95, 96], it is challenging to establish a straightforward correlation. Nevertheless, the difference between this odd δ^13^C value and the values for the BA samples in Room 1 and Room 2 paves the way to assume a shift in the mother’s diet.

This difference might be attributed to the consumption of both marine resources and C4 plant food, both bringing out such an effect on the δ^13^C isotope values but might also be linked to some specific breastfeeding-related intake. The δ^13^C value for LS2176 falls outside the range established for consumers of terrestrial resources based on faunal remains and ranks near those of available marine resources [82, 83, 97] and C4 plant consumers. The closest seashore from the La Sassa cave is at least 15 km away, with the Monti Ausoni in between. Even though we cannot exclude somewhat exploitation of marine food from the coastal strip with its lagoons and lakes, it is implausible that shellfish—rather than seafood—represented a major food for the toddler. Furthermore, the low δ^15^N value is more consistent with a diet primarily based on the C4 plant. Even accounting for the well-known increase in δ^15^N value due to breastfeeding [95, 96, 98], we should assume that an even lower δ^15^N value characterized the mother, rendering a substantial consumption of marine fish even more unlikely. The Bayesian reconstructions of the dietary preferences are consistent with this interpretation (S5 Fig in S1 File).

Unfortunately, to the best of our knowledge, there is no data on isotope values for C4 plants from Italian prehistory. Still, some data are available for *Setaria italica* and *Panicum miliaceum* from Switzerland, showing enriched δ^13^C values ranging from -10.8‰ to -9.4‰ [99]. We cannot establish whether the value recorded for LS2176 is to be attributed to a (maternal) diet that is exclusively based on the consumption of C4 plant food or only partially. However, Italian BA communities that were convincingly considered to be based on stable C4 plant exploitation [11, 12, 33], display carbon isotope signatures comparable to those of LS2176 (δ^13^C = -14.9 ± 1.1‰ at Olmo di Nogara, -15.2 ± 2.4‰ at Bovolone, and 15.4‰ at Dossetto di Nogara) [59], and the less negative values for Misa cave (δ^13^C = -16.5‰) [11] concerns people dating from the MBA.

**Fig 5:**
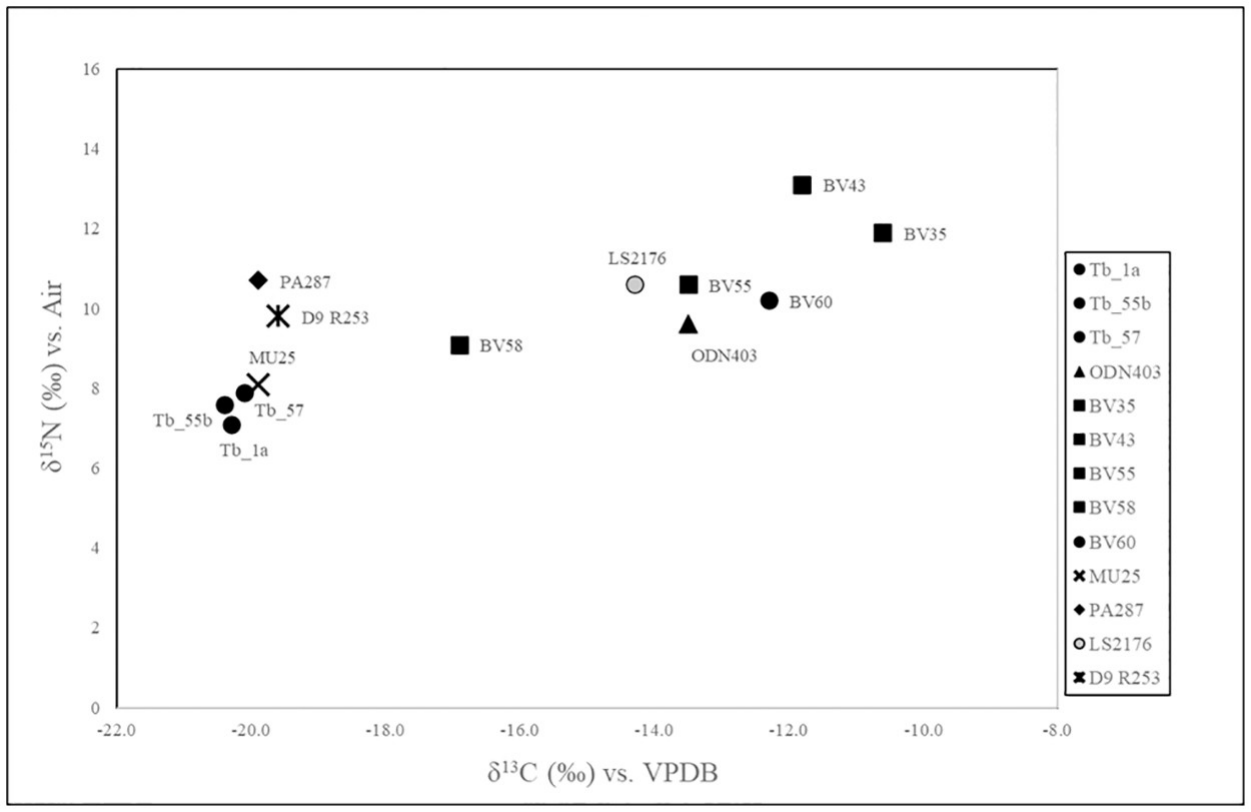
δ13C and δ15N values of infants (0–2 years) from Bronze Age (BA) Italian sites [6, 10, 53, 59]. Tb: Arano di Cellore; BV: Bovolone; MU: Monte Orcino, D9 R253: Regina Margherita; PA 287: Pastena; ODN: Olmo di Nogara.

Lastly, to identify eventual breastfeeding-related effects, we compared LS2176 data with those from Italian BA infants (approximately 0–2 years) that were already published [6, 10, 53, 59] (Fig 5). The results show differences between the toddler living in sites with C4 plants intake and the others.

LS2176 lies among children from Olmo di Nogara and Bovolone [59] rather than infants from Regina Margherita and Pastena [6, 10], which are only a few kilometers away from the La Sassa cave. Although we cannot rule out a specific mother’s diet, this evidence does not support a specific diet for breastfeeding in Latium sites during the BA. The similarity between LS2176 and the infants from Olmo di Nogara and Bovolone—where previous surveys already identified the consumption of C4 plant [59]—enhances the hypothesis related to the diet-shift towards C4 plant exploitation occurring in Latium BA. The recovery of further coeval individuals could help strengthen the assumption that the L2176 and maternal diets were C4 plant-derived. At the same time, it is also clear that all evidence strongly suggests that this infant represents the first documented individual with a C4 plant-derived diet in the Pontine area during the BA.

Whatever the precise composition was of the primary food consumed by this infant (and his/her nurse), it is remarkable that people buried in the La Sassa cave in the late phases of its exploitation had a diet that differed from that of the earlier communities. The possible introduction of this novel diet in the Pontine plain postdates its spread in Central Italy as observed in the remains from the Misa cave [11], which is consistent with a spatial and chronological trend in the dispersal of the C4-based diet from North-Eastern Italy [59] to the Southern peninsular areas. Unfortunately, its spread further South cannot yet be traced because of the lack of isotopic evidence from more southern sites.

Remarkably, the southward dispersal of the C4-based diet is consistent with the genomic and cultural evidence. Indeed, human groups with a different genetic makeup moved through the Pontine plain and the surrounding areas in the early BA, as demonstrated by the differences in genomic characteristics between CA people buried in the La Sassa cave and BA individuals from the neighbouring Grotta Regina Margherita [13]. Similarly, original stylistic influences from the North are recorded in the La Sassa ceramic assemblage for the first time in the MBA (Chapter 8 in S1 File).

The comparison of the ^87^Sr/^86^Sr ratios of the samples from the CA with those from the EBA shows that no significant differences exist in the composition of the teeth enamel. However, the variability in their ratios increases in the EBA. Conversely, Sr isotope ratios for the bones differ significantly (median CA_teeth_: 0.7091, IQR CA _teeth_: 0.0004; median EBA _teeth_: 0.7089, IQR EBA _teeth_: 0.0006; median CA_bones_: 0.7083; IQR CA_bones_: 0.0002; median EBA_bones_: 0.7085; IQR EBA _bones_: 0.0003; Mann-Whitney U = 49.5; p = 0.24 for teeth enamel and U = 25 p = 0.042 for bones), suggesting slight variations in the mobility pattern over the various periods.

Overall, EBA Sr isotope ratios are more scattered than CA ratios, in line with the few isotopic studies on the mobility of Italian prehistoric communities indicating that they became more mobile starting from the BA [8, 33, 34]. However, such an explanation is too straightforward, and the actual trend was probably more complex.

Since people in the La Sassa cave had a terrestrial diet with a moderate protein intake, the Sr introduced in their bodies only partly reflects the fingerprint of the plants/soils and is additionally related to that of the available drinking water [57, 100]. Correlations between the ^87^Sr/^86^Sr ratio of soils and those for plants have been reported in several studies [101, 102], but those for drinking water are scarce. Thus, we determined the local isotopic baseline analysing the isotope compositions of water, soil samples and fauna, including the recovered microfauna, in the ca. 78 km^2^ large area around the cave (corresponding to ca. 5 km-radius area from the cave).

**Fig 6:**
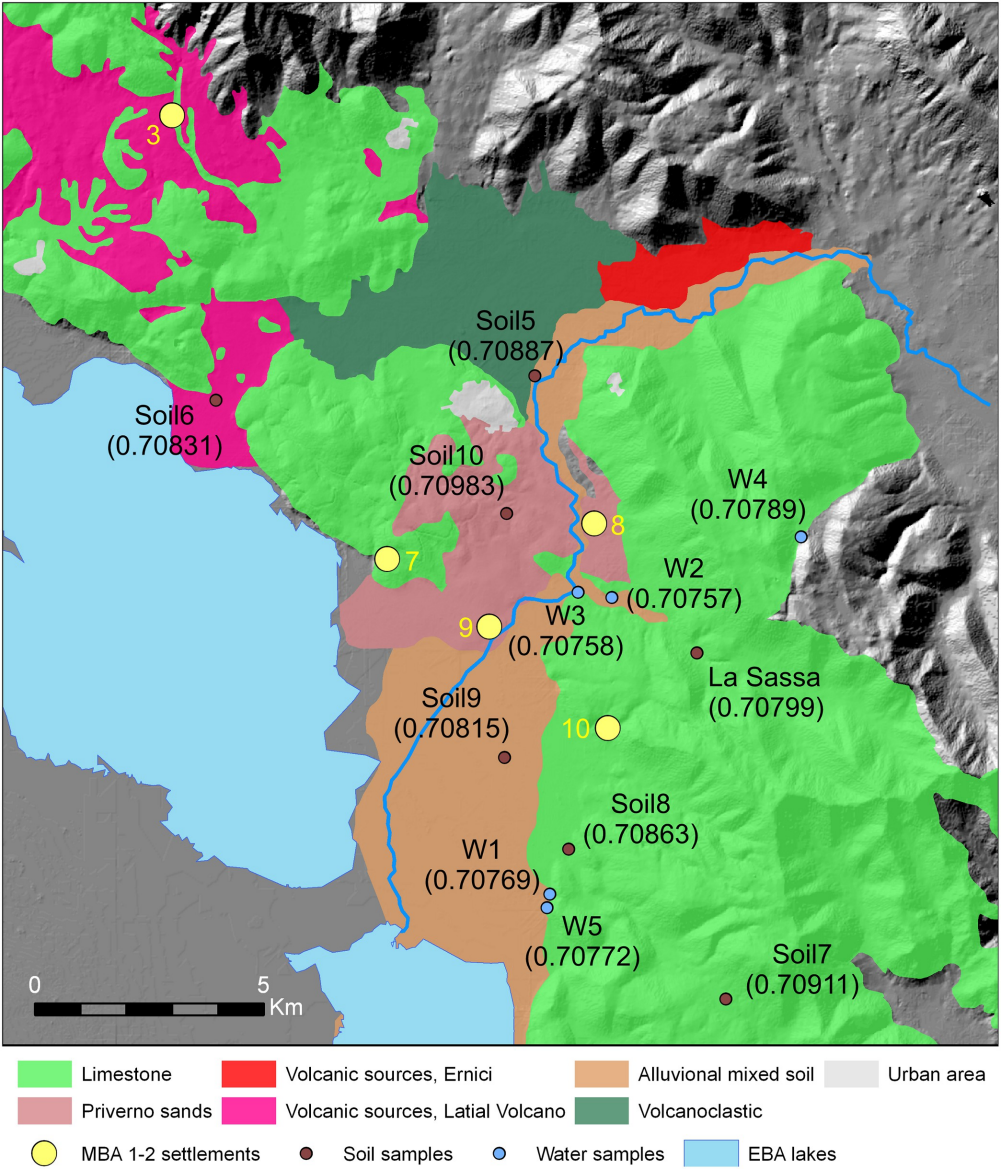
Major rock and soil types around the La Sassa cave, and sampling sites. For the settlement numbers see Fig 1. Background DEM from TINITALY/01 [21], published with a CC BY 4.0 license. W stands for Water sample.

Fig 6 shows the dominant soils and their Sr isotope ratios (S2 Table in S1 File). The ^87^Sr/^86^Sr ratios of the baseline range from 0.7075 to 0.7098.

**Fig 7:**
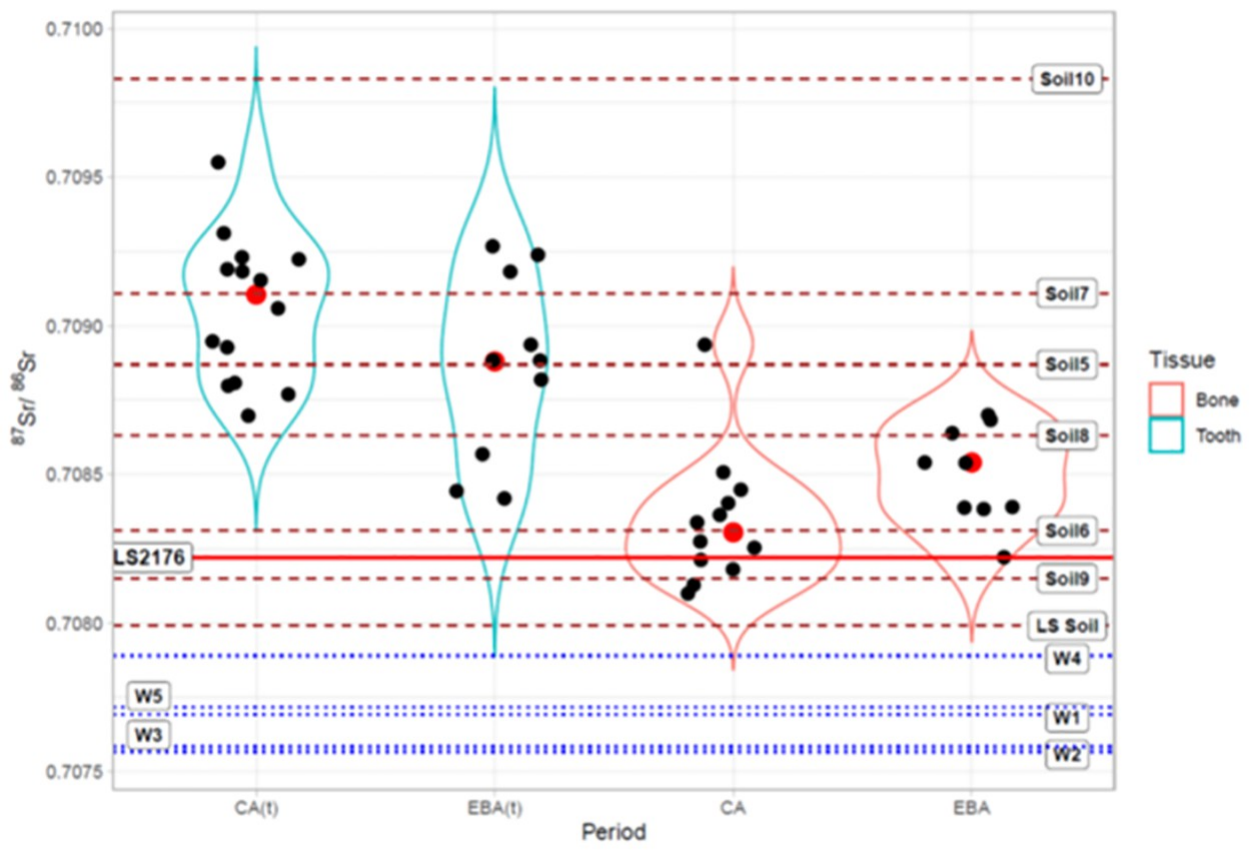
Violin plot for the Sr isotope ratios (green for teeth (t), brown for bones) for CA and EBA individuals. LS2176 is represent by a continuous red line. Black dots represent single samples, red dots are the medians. The dashed lines represent the soils, while the dotted lines indicate the water samples.

The lowest ratios are those measured on aliquots of water nowadays occurring around the La Sassa cave. However, thermal waters in the aquifers of the Monte Massico ridge, to the south of the Monti Aurunci, are marked by a variable Sr ratio, ranging from 0.7080 to 0.7096 [103]. The highest ratios have been obtained for soils with varying tephra-holding topsoil and with a large component of aeolian sands. Comparison of the isotopic signature for humans with that for the local baseline, leads to far from straightforward indications in terms of the geographical origin of both the CA and the EBA communities due to the widely varying isotopic ratios of the soils. Moreover, the overall difference in ^87^Sr/^86^Sr ratios between teeth and bones might suggests a certain degree of mobility for people between childhood and adulthood (Fig 7). Therefore, people buried in the La Sassa cave could have spent their childhood, and eventually their adulthood, in areas surrounding the cave. Regardless of the difference between teeth and bone values, most of the bone samples are enriched in radiogenic Sr with respect to the cave soil, suggesting a very small impact of secondary processes on the chemical structure of the human remains. This seems to be consistent with the fact that some individuals show teeth and bones with similar Sr isotope ratios (Fig 7) either relatively low or high.

Overall, it seems clear that slightly different geographic origins have to be supposed for those individuals and that the cave was used as a cemetery by people from many villages. This evidence is supported by radiocarbon dates spanning almost 500 years (at minimum). Indeed, to the best of our knowledge, no contemporary Central Italian CA/EBA settlement has ever been found with such a long attendance.

Looking at the distribution of the isotope composition of soils, their translation into a mobility pattern is further complicated by the question of whether the remains of the CA and EBA individuals were 1) from multiple and synchronic communities settled in a stable occupational pattern and sharing the environment around the cave, or 2) from specific, less sedentary communities living and shifting in a larger area but using the cave over a more extended period.

For the MBA, the situation is slightly different as the Sr isotope ratio for the single individual (LS2176) is rather like those for soils in the vicinity of known settlements (S5 Fig in S1 File), even though the paucity of the MBA human remains in the La Sassa cave prevents further speculations.

Even when considering a single period–CA or EBA–, the variation in the Sr isotope values around the median value is considerable (Fig 7), implying that only some buried individuals may have shared the same geochemical environment and/or may represent family groups, as already detected by genomics [13] We do not know the selection criteria for people that were buried in the La Sassa cave, but age and sex clearly were not the leading ones, since of both sexes adults and children were buried therein. From that perspective, kinship may have been one of the factors which might also explain the incidental clustering of ^87^Sr/^86^Sr values for some individuals [13].

Although we are confident that the analytical evidence properly supports our interpretations, we would explore some of the study’s limitations.

One conceivable and significant limitation of this study is the potential for selection bias. Even though grounded on a scientifically sound approach [68, 69, 104], individual identification may introduce a bias in the sample composition. We pursued a strictly conservative approach considering only the recurrent bone fragments to be sure not to overestimate the sample size for the diet reconstruction.

We did not randomly choose the bones but grounded our selection of the osteological and topographical evaluations. Even though we performed the formal analysis on the MNI, we are confident that the data on the 46 individuals reported in S4 Table in S1 File should approximate the community(ies) size. Accordingly, we also reported the data for the extended sample, which are consistent for the overall interpretation.

Indeed, the apparently restricted sample size may influence the overall analysis limiting the ability to make broader conclusions. However, the available data for the area in the chronological transect we are focusing on allow for identifying the changes at the community level in very few publications. So far, our diachronic analyses, although relying on 18 individuals (not a negligible sample size for multi-isotope analyses of prehistoric communities [6, 9, 12, 53, 85]), mark a novel approach for the area, making the evaluation not a geographically restricted assessment, instead representing a crucial step in the CA/BA knowledge supporting the recent hypothesis about the movement of BA communities [13], carrying ideas, bio-cultural characteristics, and even genetic components, from Central Europe into the Italian Peninsula.

We also recognize that several studies leveraged the present-day plant samples to establish the local biologically available strontium baseline [105–107] instead of the soil and water values. Due to logistic strategies, we preferred sampling soil and water specimens as the 87Sr/86Sr ratio of soils and those for the grown plants have been proven to be linearly correlated [101, 102]. Moreover, soil samples have been selected by considering the chart of the soils and the geological differences around the cave (See S1 File).

In seeking for potential biases, we have also analysed the faunal remains found in the cave, attributed to some species roaming around the area in the Copper/Bronze Age, to detail the environmental baseline.

Based on the wide variability displayed by the soils, even much more extensive when considering water samples, and the variation of enamel teeth, well within the soil and the faunal variability, we are confident that we considered a reliable/realistic baseline, that is consistent with the recently developed isoscapes [87], improving their resolution at a finer scale.

Another limitation could relate to the potential bias of the Sr signature of the bone and teeth due to post-deposition changes leading to an altered isotopic signature. This process could happen in the occasions of 1) pore-filling by secondary minerals; 2) recrystallization or remineralization of hydroxyapatite; 3) direct exchange with Sr in the original hydroxyapatite crystals; and 4) absorption in microcracks or onto the surfaces of original hydroxyapatite crystals [108].

However, the infrared data do not identify secondary minerals nor consider recrystallization or remineralization of hydroxyapatite. Thus, according to our FTIR analyses, we can exclude major contamination processes.

Moreover, if secondary processes occurred, one would expect alteration to have a strong ’homogenizing impact,’ obliterating any differentiation based on causes which don’t have anything to do with such diagenesis/post-depositional processes. Our data suggest something else.

We analysed the soil in the cave for comparative purposes. Such soil is strongly influenced by local water circulation in the cave and, in turn, by the possible precipitation, in recent times, of secondary minerals and elements such as Ca and Sr. However, we demonstrated that most of the bones found in the cave are enriched in radiogenic Sr than the soil and have differential signatures with respect to the teeth.

So far, even though we have acknowledged that the post-depositional changes should be assumed as a potential confounding factor, there are no obvious indications of significant diagenetic alterations in the results from the analytical techniques. Accordingly, we would wait for future studies about the in-depth exploration of the role of the post-depositional alterations through a well-defined strategy, e.g., integrating Sr data with X-ray diffraction, FTIR, scanning electron microscopy (SEM), and energy-dispersive X-ray spectroscopy (EDS), allowing for the thorough analysis of sample surfaces.

Finally, we would stress that the potential evidence of many individuals buried in La Sassa cave, either from the CA or BA, spending their childhood away and adulthood in nearby locations is the more parsimonious interpretation. We are aware that different dietary habits could also lead to that interpretation. However, this is the reason why we have previously dissected the diet of the individuals from La Sassa, following the suggestion provided by Lahtinen et al. [57], recommending the integration of either diet and mobility isotopic markers to gain a reliable reconstruction of those bio-cultural dynamics.

## Conclusions

The La Sassa cave was a funerary and cultic shelter for a long time, starting from the CA up to the MBA, and offered the opportunity to investigate the CA-BA transition in Italy by analyzing people from the same site. The human bones, faunal remains, and artifacts allow for evaluating the contemporary population dynamics in the Pontine plain and adjacent Monti Lepini and Ausoni. The isotopic data and the radiocarbon dates suggest that several CA and EBA communities have used the cave. CA and EBA individuals subsisted on slightly different diets, even though both were dominantly composed of terrestrial C3 plant-derived food. However, one individual testifies for a different diet in the late phase (MBA 2) of cave exploitation, probably linked to the southward spread of C4 plants in Central Italy. To the best of our knowledge, the evidence from the La Sassa cave is the southernmost isotopic mark for the spread of the C4 plant food consumption in the Italian BA. So far, the stylistic traits of the ceramic finds in the area indicate an increased cultural exchange from the north starting from the MBA. Thus, our isotopic data are consistent with the pottery typological analysis and support the hypothesis that the Pontine plain area was the stage for complex population dynamics at the CA/BA boundary, which could have resulted in a substantially different subsistence strategy during the MBA. The reliable connection with the demic diffusion of allochthonous people in the Central/Southern areas of the Italian peninsula contributes to the fine-mapping of dynamics leaving bio-cultural traces in the area, allowing for defining these human groups’ arrival times.

## Supporting information

S1 File (DOCX)
