# Supplementary material for: La Sassa cave: Isotopic evidence for Copper Age and Bronze Age population dynamics in Central Italy
Source: PLoS One. 2023 Jul 26;18(7):e0288637. doi: 10.1371/journal.pone.0288637 (PMC10370757; doi:10.1371/journal.pone.0288637)
Supplement: S1 File — (DOCX) [file pone.0288637.s001.docx]

**La Sassa cave: isotopic evidence of Copper Age and Bronze Age population dynamics in Central Italy.**

Marco Romboni^1,2¶^, Ilenia Arienzo^3¶^, Mauro Di Vito^3^, Carmine Lubritto^4, 5^, Monica Piochi^3^, Maria Rosa Di Cicco^4^, Olga Rickards^1^, Mario Federico Rolfo^6^, Jan Sevink^7^, Flavio De Angelis^1,8&*^, Luca Alessandri^9,10&*^

^1^ Centre of Molecular Anthropology for Ancient DNA Studies, Department of Biology, University of Rome “Tor Vergata”, Italy

^2^ Department of Biology, University of Pisa, Italy
^3^ National Institute of Geophysics and Volcanology, Vesuvius Observatory, Naples, Italy
^4^ Dipartimento di Scienze e Tecnologie Ambientali Biologiche e Farmaceutiche (DISTABiF), Università degli Studi della Campania “Luigi Vanvitelli”, Caserta, Italy
^5^ INFN Sezione Naples – CHNet
^6^ Department of History, Culture and Society, University of Rome “Tor Vergata”, Italy
^7^ Institute for Biodiversity and Ecosystem Dynamics (IBED), University of Amsterdam, Amsterdam, The Netherlands
^8^ Department of mental, physical health and preventive medicine, University of Campania Luigi Vanvitelli, Naples, Italy
^9^ Groningen Institute of Archaeology, University of Groningen, Groningen, The Netherlands

^10^ Department of Science of Antiquity, University of Rome La Sapienza, Rome, Italy

^¶^ These authors contribute equally to this work
^&^ These senior authors contributed equally to this work.

* l.alessandri@rug.nl (LA); flavio.de.angelis@uniroma2.it (FDA)

Table of contents

[**La Sassa cave: isotopic evidence of Copper Age and Bronze Age population dynamics in Central Italy.** 1](#_Toc133237388)

[1.1 Geological framework 3](#_Toc133237389)

[Fig. S1 3](#_Toc133237390)

[Table S1 4](#_Toc133237391)

[1.2 Major types of rocks, soils and parent materials in and nearby the Monti Ausoni 4](#_Toc133237392)

[1.2.1 Introduction 4](#_Toc133237393)

[1.2.2 Description of the major types of rocks and soils 5](#_Toc133237394)

[Table S2 7](#_Toc133237395)

[2 Strontium isotope results, human bones, and fauna 8](#_Toc133237396)

[Table S3 9](#_Toc133237397)

[3 FTIR results and indexes 10](#_Toc133237398)

[Fig. S3: 11](#_Toc133237399)

[Fig. S4 12](#_Toc133237400)

[4 Carbon and Nitrogen isotope results 13](#_Toc133237401)

[Table S4: 13](#_Toc133237402)

[Fig. S5 14](#_Toc133237403)

[Table S5: 20](#_Toc133237404)

[Table S6 21](#_Toc133237405)

[Table S7: Coupled Mann-Whitney test between Bronze Age Italian sites for δ13C and δ15N values. Significant p values are reported in Bold. 22](#_Toc133237406)

[5 Mobility patterns 23](#_Toc133237407)

[Fig. S6 23](#_Toc133237408)

[6 Radiocarbon dates 24](#_Toc133237409)

[Table S8 24](#_Toc133237410)

[7 References for chapters 1-6 25](#_Toc133237411)

[8 Potsherds from the sounding SP 27](#_Toc133237412)

[Fig. S7 27](#_Toc133237413)

[Fig. S8 28](#_Toc133237414)

[Table S9 30](#_Toc133237415)

## 1.1 Geological framework


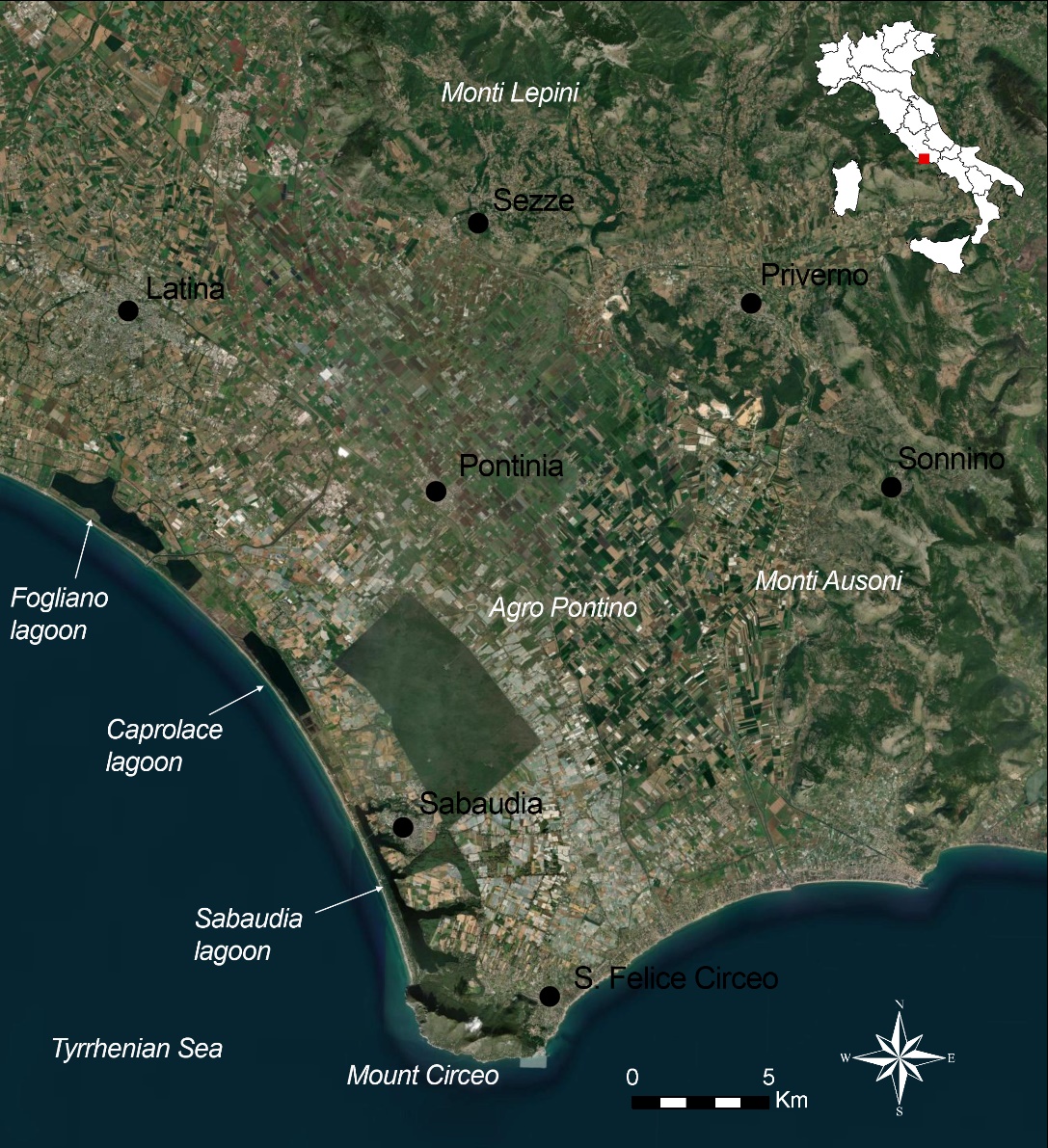
Fig. S1**:** **the study area.** Black dots, modern towns. Background from Esri, Digital Globe, GeoEye, Earthstar Geographics, CNES/Airbus DS, USDA, USGS, AeroGrid, IGN and the GIS User Community.

The La Sassa cave is situated in the west of the Monti Ausoni, which form part of the Lazio-Abruzzi Mesozoic carbonate platform of Central Italy (Fig. S1). These mountains consist of thickly bedded, lithologically quite homogenous limestones (units PC^1^C^5^ and underlying C^6-1^) dating from the Cretaceous to Paleocene (Carta Geologica d’Italia, sheet 159, Frosinone). Dolomitic limestones or dolomites are encountered in the basal strata (unit C^6-1^), which are exposed in the east, whereas in the Sonnino area limestones dominate.

The northern portion of the Monti Ausoni is bordered by a series of ca. E-W elongated tectonic basins that form the Amaseno river catchment and have a varied geology. In the west, further downstream, the Amaseno first runs through a narrow valley in between the Monti Ausoni and aeolian sand-covered limestone hills of Priverno/San Martino and then enters the Agro Pontino graben. In this graben, the Mesozoic carbonate platform is encountered at considerable depth below sea level, due to the presence of large faults: A roughly NW-SE running fault, bordering the Monti Lepini and a N-S running fault, bordering the Monti Ausoni in the west. In the Agro Pontino, Holocene deposits prevail, with some outcrops of older, lagoonal deposits of Eemian age (the Borgo Ermada marine complex^1^), and of alluvial fans and debris slopes descending from the Monti Ausoni.

Whereas the geological map and associated studies of the basal rock geology provide adequate information on the earlier geological formations^2^, detailed geological studies of the Quaternary deposits are scant and largely limited to the Ernici volcanism, later renamed as Volsci Volcanic Field (south of the area, outside of Fig. 1). Results of the study of this volcanism have been summarized by Peccerillo ^3^ and Marra et al. ^4^. Information on the distribution and composition of the Quaternary deposits can be found in the soil and landscape studies by Sevink et al.^1^ and Arnoldus-Huyzendveld et al.^5^. Together, this information allowed for the identification of types of rocks and soils that may differ with respect to their isotopic composition and are relevant for our study. An overview of the most relevant soils is given in Table S1, while a full explanation is presented in Chapter 1.2.

| Soil sample label | Nature of material | Significance |
| --- | --- | --- |
| Soil5 | Recent river sediment from near Priverno in Amaseno valley | Representative for Amaseno sediment upstream of Priverno, with large Mti. Lepini component |
| Soil6 | Tuffaceous bed intercalated in alluvial fan deposit with coarse limestone gravels | Representative for Mti. Lepini derived sediment with high tephra component. |
| Soil7 | Soil from in between tower karst at Campo Soriano | Representative for deep limestone weathering soils with more or less tephra-holding topsoil |
| Soil8 | Deep Vertisol with strong slickensides and abundant Mn mottles | Representative for old terra rossa soil, dominantly derived from limestone Mti. Ausoni |
| Soil9 | Recent Amaseno sediment, mixture of all kinds of materials, including Pleistocene aeolian deposits | Representative for truly mixed sediment of Amaseno river. |
| Soil10 | Middle Pleistocene Priverno sands with deep paleosol | Representative for large complex of aeolian sands of Priverno |

Table S1: **major types of soils and parent materials in and near the Monti Ausoni**

## 1.2 Major types of rocks, soils and parent materials in and nearby the Monti Ausoni

### 1.2.1 Introduction

Within the context of this study of the human remains in the La Sassa cave, relevant questions are:

A) What types of rocks and soils are encountered in the vicinity of the La Sassa cave that may have resulted in specific Sr-isotopic signatures of the vegetation on these rocks and soils, and directly or indirectly determined the Sr-isotopic signature of food ingested by inhabitants of the area, of which remains were retrieved in the cave?

B) What variation in isotopic composition of the water from karstic springs can be expected, given the geology of the area?

Whereas the variation in rocks and soils is considerable (Table S2), as will be described later, the water from karstic springs likely has a very uniform composition, the dominant geological formation being the limestones of the PC1C5 unit. It is in this unit that the karstic system is developed and from which karstic sources spring on the lower slopes and in the Amaseno valley, as is evident from the geological map. This description therefore is focused on the types of rocks and soils in relation to question A. Quite evidently it can be limited to the dominant types of rocks and soils in the vicinity of the La Sassa cave, since very local or rare types with potentially deviating Sr-isotopic signatures are very unlikely to have had a significant impact on the overall isotopic signature of the ingested food.

### 1.2.2 Description of the major types of rocks and soils

Extensive information is available on the rocks and soils in the area of study, in the form of a 1:100.000 geological map (sheet 159, Frosinone) and of a soil map at scale 1:100.000 by Sevink et al. ^1^. A modified version of this latter map has been published by Arnoldus-Huyzendveld, Perotto and Sarandrea ^6^. Both maps are available online (http://sgi.isprambiente.it/geologia100k/mostra_foglio.aspx?numero_foglio=159; I SUOLI DELLA PROVINCIA DI LATINA https://www.provincia.latina.it › flex). Major rock and soil types are described below and depicted in Fig 4.

- Limestones (samples Soil7 and Soil8).

The lithology of the limestones is very uniform. They are very pure and hold low amounts of non-carbonate clastic material less than a few percent ^7^, implying that even upon prolonged weathering and dissolution only a limited amount of residual clastic material will accumulate. Nevertheless, the common occurrence of deep terra rossa soils is typical for the Monti Ausoni, as demonstrated by the soil map (units M1e-k, M2 and M3), and they are often associated with pronounced karstic features such as at Campo Soriano. Thus, terra rossa soils, whether in situ or more or less colluviated and occurring as fill in karstic depressions, form an important substrate for the vegetation, rather than the hard and dense limestones.

Though at first sight being a weathering residue of limestone, aeolian dust and volcanic tephra are described as important components of such terra rossa soils throughout the Central Mediterranean ^7–11^. This was extensively demonstrated for the Southern Lazio by Spaargaren ^7^, who showed that its terra rossa soils commonly hold a significant amount of volcanic tephra, which may take the form of a distinct thick top stratum of strongly weathered tephra of which all but the most resistant minerals have disappeared. In the area of study, tephra from the Colli Albani (south Latium, outside Fig. 1) dominates, evidenced by its abundant presence in the hills to the W of the Amaseno basin, but part of the tephra may originate from the much smaller eruption centers of the Volsci Volcanic Field, such as the Guiliano di Roma volcano. It is not excluded that the terra rossa soils contain some non-volcanic aeolian material, since massive Pleistocene aeolian sands are encountered in between Fossanova and Priverno (units e1, geological map), but in the regional soil studies cited such component is not described. Nevertheless, these terra rossa soils are likely to hold some silt-size or finer aeolian material of African origin, which has been reported in many studies on terra rossa soils  ^12–14^ in Italy and adjacent countries.

In summary, distinction has to be made between the limestones and the terra rossa soils, whether in situ or reworked to form alluvial cones and debris slopes (units qr), since this terra rossa material is typically partly allochthonous. This was demonstrated by the analysis of material filling the La Sassa cave and other caves in the Sonnino area, which were found to contain sand-size pyroxene, black garnet and sanidine crystals, which considering their size most probably are from the Volsci volcanic field. However, other than in the Monti Lepini (Figure S1), in the Monti Ausoni nowhere recognizable tephra layers were encountered in these terra rossa soils.

- Other pre-Quaternary rocks

In the middle reaches of the Amaseno river catchment and in the Ausoni mountains (near Monte Alto) some outcrops occur of Late Miocene fine-grained sedimentary rocks, described as “argille grigie o varicolori (units ce) and holding olistoliths of older rocks (mostly limestones). On the soil map they are depicted as units L. Though their distribution is limited, these rocks are relevant, being one of the rare sources of non-carbonatic clastic material in the Amaseno catchment.

- Pyroclastic rocks of the Volsci Volcanic Field and associated sedimentary deposits.

The major eruption center is that of Guiliano di Roma, associated with its basalt outcrop (unit R4 on the soil map). Far more widespread are tephra from this volcano and other eruption centers, generally depicted as units R1 on the soil map. They are quite abundant in the northern part of the Macchia di San Stefano tectonic basin, particularly in the valley of the Fiume Monteacuto, NW of Guiliano di Roma. Their most western occurrence is immediately north of Roccasecca in the Priverno basin, where lithoid tuff is encountered (units R3a) intercalated in contemporary lacustrine deposits. A nearby local eruption center produced tephra (unit R1a) holding large pyroxene, sanidine and garnet crystals (up to 1 cm). The Volsci tephra were an important sediment source for the later Amaseno river deposits. The various volcanic rocks are relatively well studied, including their Sr-isotopic composition, though within the area of study, the attention is rather focused on the Guiliano di Roma volcano ^3^.

- Pyroclastic rocks from the Colli Albani (sample Soil6).

Tephra from the Colli Albani volcano are particularly encountered in the Monti Lepini, N of Priverno and were erupted over a fairly long period i.e. the whole of the middle Pleistocene. Thus, near Sezze, presumed Colli Albani tephra completely cover and hide the underlying karstic limestone relief. Colli Albani tephra also abound in the Carpineto Romano valley and extend far into the large valley descending towards Roccagorga-Maenza, where it is encountered as distinct tephra layers in the large older alluvial fans (units C1k). The same situation is encountered in the west of the Priverno basin ^1^, where near Colle Rotondo and Ceriara tephra layers abound in the alluvial fans descending from the Monti Lepini into this basin (units C1f) and subsequently into the Agro Pontino. Remarkable is that such tephra is far less common in the aeolian sand covered limestone hills west of Priverno, where tephra layers were only incidentally encountered ^1,15^. The stratigraphy and geochemistry of the Colli Albani pyroclastic rocks have been extensively studied^16,17^.

- Pleistocene aeolian sands of Priverno (sample 10).

The hills to the west and south of Priverno are thickly covered by highly quartzitic aeolian sands that were deposited in several phases as evidenced by the occurrence of intercalated paleosols and (rare) tephra layers. They are deeply weathered and have a very specific mediterranean cork oak (*Quercus suber*) vegetation, having completely decalcified, acid soils (units g5-6, soil map). They are underlain by Middle Pleistocene fossiliferous lacustrine clays (units H5) that in their turn cover the limestone basement, which is exposed in the valley floor. The origin of the aeolian sand has to be found in long-shore NW-SE directed sediment transport along the Tyrrhenian coast during the Early and Middle Pleistocene, with the Tiber catchment or even further north lying basins as major source area of the sand. An important feature is that the sand is very low in volcanic minerals ^1,15^, suggesting that its transport took place prior to significant volcanic activity of the Colli Albani or other volcanoes in Northern Lazio, i.e. prior to the Middle Quaternary ^16^.

- Alluvial deposits of the Amaseno river and its tributaries (samples Soil5 and Soil9).

The Amaseno river and its tributaries derive their sediment from a quite large range of rocks, including Mesozoic limestones, Tertiary clastic rocks, tephra from the Colle Albani and Ernici group, Middle Pleistocene lacustrine deposits, and, downstream of Priverno, highly siliceous Early to Middle Pleistocene aeolian sands. The deforestation and massive land use that started in Early Roman times, evidently had a major impact on the fluvial system, leading to massive input of eroded soil material and an equally massive accumulation of sediment in the inner valley and built up of a large alluvial fan in the Agro Pontino, where the Amaseno enters this basin (unit E1c, soil map). Inland, this is evidenced by the presence of a thick relatively fine-textured and homogenous layer of sediment, covering a fluvial system with more varied sediments. This thick ‘colluvial’ layer upslope grades into similar slope deposits and alluvial cones, sometimes with intercalated gravelly layers formed by incidental flash floods. It is indicated as unit A1m, when truly fluvial, and as units E5-6 when more colluvial. In the latter case, the composition is more varied, because of the local geology and soils. On the geological map the deposits are indicated as an undifferentiated unit a2.

During most of the Middle Holocene, a large coastal lake existed, formed by inundation of the deeply incised Amaseno valley. Around 2000 BC, sea level stabilized at a level which was slightly below the current sea level and in this lake the Amaseno river built up an alluvial fan. This fan blocked the outlet of the inner part of the Agro Pontino graben, which led to the development of a second inland lake, of which the origin and dimensions have been reconstructed in rather detail^18,19^. Though the Amaseno river gradually expanded this alluvial fan southward, the lake persisted till Early Roman times ^20^. The alluvial fan is indicated as unit E1c.

| **Sample** | **UTM WGS84** | **UTM WGS84** | **Analysed material** | **^87^Sr/^86^Sr** |
| --- | --- | --- | --- | --- |
|  | **EAST** | **NORTH** |  |  |
| W1 (Marutte) | 349484 | 4582365 | Water | 0.70769 |
| W2 (Bagnoli) | 350841 | 4588855 | Water | 0.70757 |
| W3 (La Fontanella) | 350099 | 4588964 | Water | 0.70758 |
| W4 (Lucerna) | 354982 | 4590182 | Water | 0.70789 |
| W5 (Fonte del Porto 2) | 349420 | 4582066 | Water | 0.70772 |
|  |  |  |  |  |
| Soil5 | 349088 | 4593497 | Soil | 0.70887 |
| Soil6 | 342116 | 4592967 | Soil | 0.70831 |
| Soil7 | 353267 | 4579866 | Soil | 0.70911 |
| Soil8 | 349825 | 4583146 | Soil | 0.70863 |
| Soil9 | 348426 | 4585149 | Soil | 0.70815 |
| Soil10 | 348468 | 4590486 | Soil | 0.70983 |
| La Sassa soil | 352632 | 4587441 | Soil | 0.70799 |

Table S2: **strontium isotope ratio from soils and spring waters around the La Sassa cave**

## 2 Strontium isotope results, human bones, and fauna

| **Sample** | **Species** | **Chronology** | **Analyzed material** | **Anatomical district** | **US** | **^87^Sr/^86^Sr** |
| --- | --- | --- | --- | --- | --- | --- |
| LS405 | *Ovis vel Capra* | CA | Faunal | Tooth | 19 | 0.70816 |
| LS421 | *Ovis vel Capra* | CA | Faunal | Incisor | 30 | 0.70825 |
| LS422 | *Ovis vel Capra* | CA | Faunal | Incisor | 30 | 0.70806 |
| LS423 | *Ovis vel Capra* | CA | Faunal | Tooth | 33 | 0.70836 |
| LS419 | *Equus sp.* | CA | Faunal | Incisor | 36 | 0.70915 |
| LS420 | *Microtus sp.* | CA | Faunal | Mandible | 36 | 0.70803 |
| LS126 | *H. sapiens* | CA | Human bone | Femur | 19 | 0.70818 |
| LS130 | *H. sapiens* | CA | Human bone | Femur | 19 | 0.70836 |
| LS151 | *H. sapiens* | CA | Human bone | Femur | 19 | 0.70825 |
| LS418 | *H. sapiens* | CA | Human bone | Femur | 19 | 0.70813 |
| LS212 | *H. sapiens* | CA | Human bone | Mandible | 19 | 0.70810 |
| LS212 | *H. sapiens* | CA | Human teeth | M_1_ Right | 19 | 0.70881 |
| LS150 | *H. sapiens* | CA | Human bone | Mandible | 19 | 0.70827 |
| LS150¹(t) | *H. sapiens* | CA | Human teeth | C_1_ Left | 19 | 0.70931 |
| LS150²(t) | *H. sapiens* | CA | Human teeth | P_4_ Left | 19 | 0.70923 |
| LS150³(t) | *H. sapiens* | CA | Human teeth | M_1_ Left | 19 | 0.70918 |
| LS115 | *H. sapiens* | CA | Human teeth | M^1^ Right | 19 | 0.70870 |
| LS209 | *H. sapiens* | CA | Human teeth | C^1^ Right | 19 | 0.70877 |
| LS285 | *H. sapiens* | CA | Human teeth | I_1_ Right | 19 | 0.70893 |
| LS1956 | *H. sapiens* | CA | Human bone | Mandible | 19 | 0.70821 |
| LS1956(t) | *H. sapiens* | CA | Human teeth | M_1_ Right | 19 | 0.70895 |
| LS120 | *H. sapiens* | CA | Human bone | Maxillae | 19 | 0.70840 |
| LS120(t) | *H. sapiens* | CA | Human teeth | M^2^ Left | 19 | 0.70922 |
| LS346 | *H. sapiens* | CA | Human teeth | C_1_ Left | 31 | 0.70955 |
| LS64 | *H. sapiens* | CA | Human teeth | M^1^ Left | 36 | 0.70880 |
| LS68 | *H. sapiens* | CA | Human bone | Femur | 45 | 0.70894 |
| LS4852 | *H. sapiens* | CA | Human bone | Mandible | 97 | 0.70845 |
| LS4852(t) | *H. sapiens* | CA | Human teeth | M_1_ Right | 97 | 0.70915 |
| LS578 | *H. sapiens* | CA | Human bone | Mandible | 55 | 0.70834 |
| LS578 (t) | *H. sapiens* | CA | Human teeth | M_1_ Right | 55 | 0.70906 |
| LS565 | *H. sapiens* | CA | Human bone | Mandible | 55 | 0.70851 |
| LS565(t) | *H. sapiens* | CA | Human teeth | M_2_ Right | 55 | 0.70919 |
| LS896 | *H. sapiens* | EBA | Human bone | Mandible | 75 | 0.70838 |
| LS896(t) | *H. sapiens* | EBA | Human teeth | M_1_ Right | 75 | 0.70857 |
| LS2203 | *H. sapiens* | EBA | Human bone | Mandible | 78 | 0.70854 |
| LS2203(t) | *H. sapiens* | EBA | Human teeth | M_2_ Right | 78 | 0.70844 |
| LS916 | *H. sapiens* | EBA | Human bone | Mandible | 78 | 0.70839 |
| LS916(t) | *H. sapiens* | EBA | Human teeth | M_2_ Left | 78 | 0.70888 |
| LS1978 | *H. sapiens* | EBA | Human bone | Mandible | 78 | 0.70839 |
| LS1978(t) | *H. sapiens* | EBA | Human teeth | M_2_ Right | 78 | 0.70894 |
| LS865 | *H. sapiens* | EBA | Human bone | Mandible | 78 | 0.70868 |
| LS865(t) | *H. sapiens* | EBA | Human teeth | M_2_ Left | 78 | 0.70927 |
| LS2208 | *H. sapiens* | EBA | Human bone | Mandible | 78 | 0.70854 |
| LS2208(t) | *H. sapiens* | EBA | Human teeth | M_2_ Right | 78 | 0.70842 |
| LS2161 | *H. sapiens* | EBA | Human bone | Mandible | 78 | 0.70864 |
| LS2161(t) | *H. sapiens* | EBA | Human teeth | M_1_ Right | 78 | 0.70924 |
| LS2177 | *H. sapiens* | EBA | Human bone | Mandible | 78 | 0.70870 |
| LS2177(t) | *H. sapiens* | EBA | Human teeth | M_3_ Left | 78 | 0.70888 |
| LS882 | *H. sapiens* | EBA | Human bone | Mandible | 78 | 0.70822 |
| LS882(t) | *H. sapiens* | EBA | Human teeth | C_1_ Left | 78 | 0.70882 |
| LS2178(t) | *H. sapiens* | EBA | Human teeth | M_1_ Right | 78 | 0.70918 |
| LS2176 | *H. sapiens* | MBA2 | Human bone | Femur | RA | 0.70815 |
| LS2176* | *H. sapiens* | MBA2 | Human bone | Femur | RA | 0.70817 |
| LS2176(t) | *H. sapiens* | MBA2 | Human teeth | I_1_Right | RA | 0.70822 |

Table S3: **strontium isotope ratios for human bones and teeth, and for fauna collected inside the La Sassa cave.** *, different aliquot of the same femur; (t), tooth


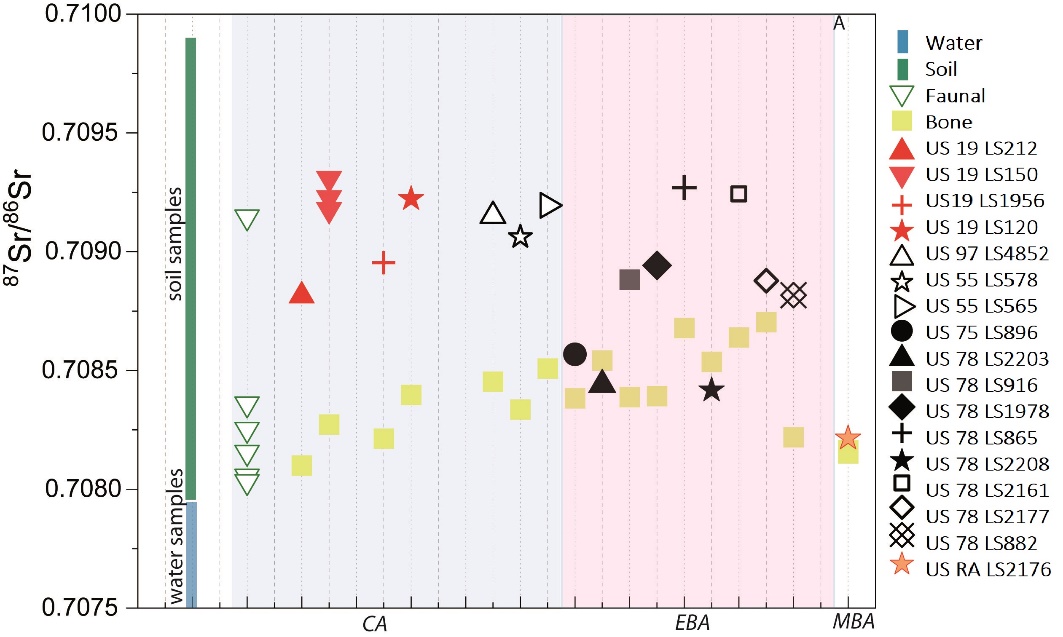


**Fig. S2: figure displays the Sr isotope compositions of the analysed soil and water samples.** These data are compared with those acquired for the enamel teeth sampled from the individuals recovered in La Sassa cave, and the corresponding bones (orange squares)

## 3 FTIR results and indexes

The spectra for the studied materials are very similar, except for the *infant* bone (Figure S3). The results indicate that the samples analyzed are low in type A carbonate because of the weak or absent absorption bands at around 1540 cm^-1^ and 868 cm^-1^ ^21–23^. Substitution of trivalent phosphate ions (type B) by carbonate ions can be unambiguously identified by strong absorption bands at around 1415 cm^-1^; these phosphate ions/such carbonate ions produce dominant bands at frequencies of ca. 1450 cm^-1^ and 870 cm^-1^ (deformational modes). Absorption bands of inorganic calcite - ν_4_(CO_3_^-2^) at ca. 712 cm^−1^ - were not encountered. Protein absorption bands occur at around 1660 cm^-1^, whereas they are less evident at 1565 and 1545 cm^-1^. Phosphate groups dominate the spectra; in all samples fluorapatite is present, revealed by the distortion at 1090 cm^-1^ ^24^. Typically, fluorapatite is preserved in fossil bones.

The infant bone differs from the other bones with respect to the carbonate and organic functional groups. The 1450-1415 cm^-1^ doublet is absent, at 1415 cm^-1^ a signal occurs and is readable, the 870 cm^-1^ peak is absent, and clear peaks occur at 1450 cm^-1^ and 878 cm^-1^, implying that carbonate A dominates in the bioapatite, although the 1545 cm^-1^ signal is not visible. These features used in study of bones growth, texture and fragility, likely should be attributed to the young age of the individual or its metabolism^25-26^. The alternative possibility of cremation can be excluded since the peaks in the region 1100 and 550 cm^-1^ are not distorted and a fluorapatite signal at 1090 cm^-1^ is present in all samples^27,28^. In contrast to the other samples, the infant bone also shows peaked absorption at ca. 711 cm^-1^ of CO(CaCO_3_) ^29^, at ca. 915 and 2875 cm^-1^ due to ν(C-N)PRO and νCH_lipids_, respectively ^30^, and at ca. 1793 and 2511 cm^-1^. We could not attribute the latter two peaks to specific compounds ^22–24,31^.

FTIR spectra lack of appreciable OH—stretching and deformation of the Si-Al-O bonds in the OH- and fingerprint regions reported in Madejova’ and Komadel^32^. Only the LS2176 presents a bands at 3694 cm-1 for which cannot be found unique correspondence with Si-O and Al-Si-O at frequency <550 cm-1 suggested by authors

Based on the PCI (crystallinity), the Amide amount (Am/P), the Carbonate/Phosphate ratio (C/P and CC/PP) and the Carbonate distribution (C(A+B)_1450_/C(A+B)_870_) two different groups of materials could be distinguished: group 1 composed of LS151, LS865, LS2208, LS418, LS2177 and LS2203, and group 2 that includes the infant tooth (Figure S4). The infant bones would belong to group 2 depending on the interpretation of the doublet absence and the 1415 cm^-1^ signal. Thus, two different trends could be distinguished in the de-proteinization and phosphatation leading to a decline of the carbonate content (see arrow in panel a). The most de-proteinized sample is LS2208 with coherent very low N content (Table 1). LS565, LS2177, LS4852 and LS2161 also are de-proteinized samples with high N content (Table 1), with the exception of LS2161. Within each group, bone samples LS151, LS2208, LS120 and LS1978 are similarly de-proteinized and phosphatized (see arrow in Fig. S4). Based on both doublet absence and BAI index, type-A carbonate strongly contributes to the infant bone^21^.


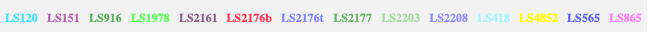

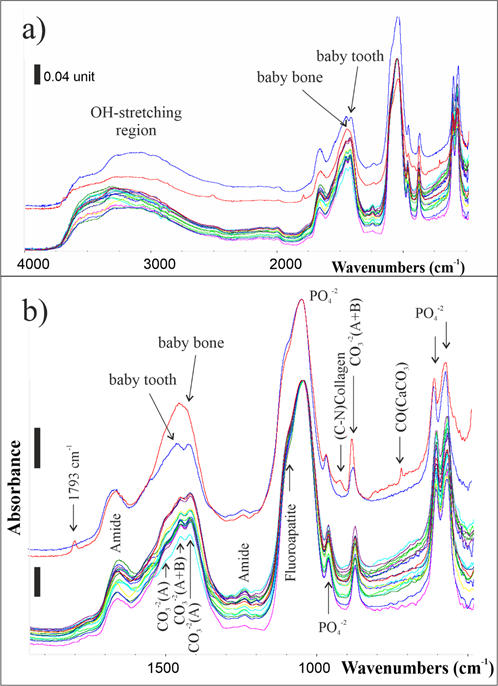


Fig. S3: **FTIR spectra of analyzed bone samples (a) and zoomed view of the indicized range between 1900 and 565 cm^-1^ (b).** The assignment of the bands is based on literature ^21–25,28–31, 33^ and can also be found in Table S6. The deviating FTIR spectra for bone remains of the LS2176 infant (baby bone in the figure) and for its single tooth (baby tooth in the figure) are specifically indicated.


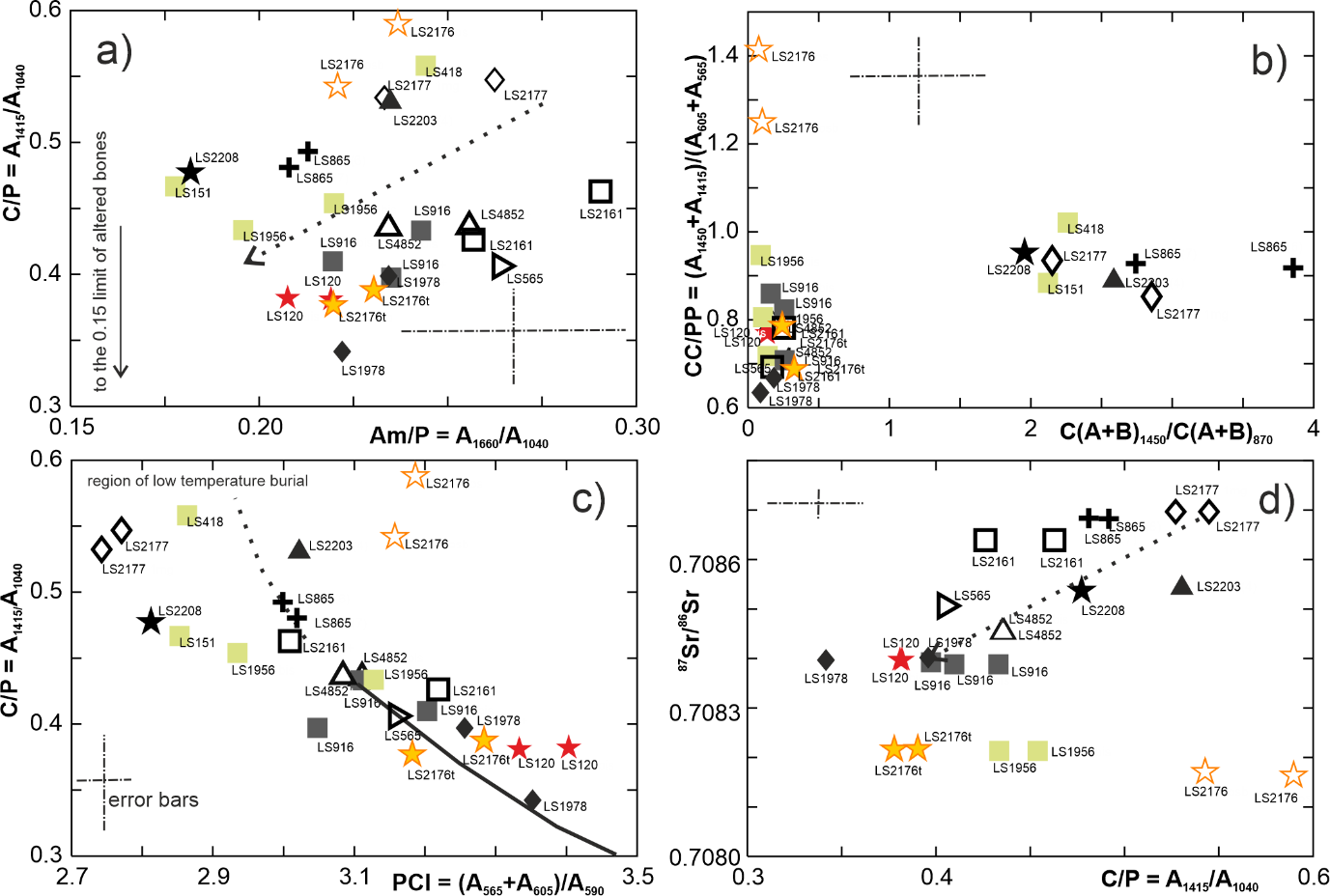


Fig. S4**: Relation between Carbonate/Phosphate ratios and Amide/Phosphate index (a), and Carbonates (b) Phosphate Crystallinity (c), and (d) ^87^Sr/^86^Sr.** The arrows in (a) indicate the general decrease of amide with increasing phosphate crystallinity. The line in (c) is the trend following Paba et al.^28^ from the low temperature (upper on the left) toward the high temperature (lower on the right) burial. The line in (d) is the decrease of Sr^87^ with Carbonate/Phosphate ratios. Same symbols for replicated analyses.

## 4 Carbon and Nitrogen isotope results

Table S4: **Stable carbon and nitrogen isotope ratios in the extended sample of human bones from the La Sassa cave.** R=Right; L=Left A; CA=Copper Age; EBA=Early Bronze Age; MBA2=Middle Bronze Age sub-phase 2.

Fig. S5**: Bayesian reconstruction through FRUITS algorithm (Fernandes et al., 2014) of the dietary preferences: (a) LS2176; (b) estimated Mother/Nurse of LS2176; (c) Differential C4 plant consumptions; (d) Differential Marine resource consumption.** The detailed parameters are specified as in Cortese et al., 2022. LaSassaCA and LaSassaEBA refer to the average values for the Copper Age and Early Bronze Age groups, respectively.

| **Period** | **Region** | **Sites** | **Samples ID** | **Species** | **δ^13^C (‰) vs. VPDB** | **δ^15^N (‰) vs. Air** | **C/N** | **References** |
| --- | --- | --- | --- | --- | --- | --- | --- | --- |
| ND | Latium | La Sassa | L.S. 481 | *Bos taurus* | -20.5 | 9 | 3.3 | This study |
| ND | Latium | La Sassa | L.S. 514 | *Sus domesticus* | -20.9 | 5.2 | 3.4 | This study |
| BA | Latium | La Sassa | L.S. 793 | *Bos taurus* | -22.5 | 5.5 | 3.3 | This study |
| CA | Latium | La Sassa | L.S. 595 | *Ovis vel Capra* | -21.7 | 4.7 | 3.2 | This study |
| BA | Latium | La Sassa | L.S. 535 | *Cervus elaphus* | -22.4 | 5.3 | 3.3 | This study |
| BA | Latium | La Sassa | L.S. 794 | *Bos taurus* | -21.8 | 5.5 | 3.2 | This study |
| ND | Latium | La Sassa | L.S. 517 | *Sus domesticus* | -21.2 | 4.9 | 3.3 | This study |
| CA | Latium | Poggio Olivastro | PO 1 | *Vulpes vulpes* | -18.7 | 8.1 | 3.3 | *Bernardini et al. 2021* |
| CA | Latium | Poggio Olivastro | PO 5 | *Ovis vel capra* | -21.5 | 5.7 | 3.2 | *Bernardini et al. 2021* |
| CA | Latium | Poggio Olivastro | PO 8 | *Bos taurus* | -19.9 | 6 | 3.2 | *Bernardini et al. 2021* |
| CA | Latium | Poggio Olivastro | PO 9 | *Bos taurus* | -21.6 | 4.9 | 3.6 | *Bernardini et al. 2021* |
| CA | Latium | Poggio Olivastro | PO 14 | *Bos taurus* | -18.6 | 6.4 | 3.3 | *Bernardini et al. 2021* |
| CA | Campania | Eboli | E F4 | *Ovis vel capra* | -20.6 | 8.8 | 3.4 | *Bernardini et al. 2021* |
| CA | Campania | Buccino | BU F1 | *Cervus elaphus* | -21.33 | 4.73 | 3.5 | *Bernardini et al. 2021* |
| CA | Campania | Buccino | BU F2 | *Sus sp.* | -20.32 | 9.44 | 3.3 | *Bernardini et al. 2021* |
| CA | Campania | Buccino | BU F4 | *Cervus elaphus* | -20.21 | 9.21 | 3.4 | *Bernardini et al. 2021* |
| CA | Campania | Buccino | BU F5 | *Ovis vel capra* | -20.58 | 8.15 | 3.4 | *Bernardini et al. 2021* |
| CA | Campania | Buccino | BU F6 | *Bos taurus* | -20.1 | 9.95 | 3.4 | *Bernardini et al. 2021* |
| CA | Campania | Buccino | BU F7 | *Ovis vel capra* | -20.97 | 8.87 | 3.4 | *Bernardini et al. 2021* |
| CA | Campania | Buccino | BU F8 | *Sus sp.* | -20.69 | 8.59 | 3.4 | *Bernardini et al. 2021* |
| CA | Campania | Buccino | BU F9 | *Sus sp.* | -20.47 | 8.69 | 3.4 | *Bernardini et al. 2021* |
| MBA | Veneto | Olmo di Nogara | ODNA | *Sus sp.* | -15.4 | 7.3 | 3.4 | *Tafuri et al. 2018* |
| MBA | Veneto | Olmo di Nogara | ODNB | *Ovis vel capra* | -16.7 | 5.4 | 3.3 | *Tafuri et al. 2018* |
| MBA | Veneto | Olmo di Nogara | ODNC | *Bos taurus* | -17.8 | 6.5 | 3.3 | *Tafuri et al. 2018* |
| MBA | Veneto | Olmo di Nogara | ODN_bos_(t.221) | *Bos taurus* | -19 | 7.2 | 3.3 | *Tafuri et al. 2018* |
| MBA | Veneto | Olmo di Nogara | ODN_bos | *Bos taurus* | -22.1 | 7.4 | 3.2 | *Tafuri et al. 2018* |
| MBA | Veneto | Olmo di Nogara | ODN(MU) | *Cervus elaphus* | -20.9 | 6.3 | 3.2 | *Tafuri et al. 2018* |
| MBA | Veneto | Olmo di Nogara | PD10 | *Ovis vel capra* | -21.6 | 7.2 | 3.2 | *Tafuri et al. 2018* |
| EBA1–EBA2 | Veneto | Dossetto di Nogara | Dossetto_bos | *Bos taurus* | -20 | 4.2 | 3.2 | *Tafuri et al. 2018* |
| EBA1–EBA2 | Veneto | Dossetto di Nogara | PD8 | *Cervus elaphus* | -20.9 | 4.9 | 3.2 | *Tafuri et al. 2018* |
| EBA1–EBA2 | Veneto | Dossetto di Nogara | PD7 | *Sus domesticus* | -20.8 | 5.3 | 3.3 | *Tafuri et al. 2018* |
| RBA1–RBA2 | Veneto | Fondo Paviani | FP6 | *Bos taurus* | -17.6 | 5.7 | 3.3 | *Tafuri et al. 2018* |
| RBA1–RBA2 | Veneto | Fondo Paviani | FP9 | *Bos taurus* | -17.9 | 7.2 | 3.2 | *Tafuri et al. 2018* |
| RBA1–RBA2 | Veneto | Fondo Paviani | FP12 | *Bos taurus* | -19.2 | 5.8 | 3.4 | *Tafuri et al. 2018* |
| RBA2 | Veneto | Fondo Paviani | FP15 | *Bos taurus* | -18.6 | 6.7 | 3.6 | *Tafuri et al. 2018* |
| RBA2 | Veneto | Fondo Paviani | FP18 | *Bos taurus* | -18.6 | 6.3 | 3.2 | *Tafuri et al. 2018* |
| RBA2 | Veneto | Fondo Paviani | FP24 | *Cervus elaphus* | -20.9 | 4.9 | 3.2 | *Tafuri et al. 2018* |
| RBA1–RBA2 | Veneto | Fondo Paviani | FP11 | *Cyprinidae* | -24.7 | 7.9 | 3.4 | *Tafuri et al. 2018* |
| RBA1–RBA2 | Veneto | Fondo Paviani | FP10 | *Esox lucius* | -20.5 | 12.7 | 3.2 | *Tafuri et al. 2018* |
| RBA1–RBA2 | Veneto | Fondo Paviani | FP3 | *Esox lucius* | -22.7 | 11.1 | 3.1 | *Tafuri et al. 2018* |
| RBA1–RBA2 | Veneto | Fondo Paviani | FP14 | *Ovis vel capra* | -18.6 | 8.3 | 3.3 | *Tafuri et al. 2018* |
| RBA2 | Veneto | Fondo Paviani | FP17 | *Ovis vel capra* | -19.5 | 8.2 | 3.2 | *Tafuri et al. 2018* |
| RBA2 | Veneto | Fondo Paviani | FP19 | *Ovis vel capra* | -19.2 | 6.7 | 3.1 | *Tafuri et al. 2018* |
| RBA2 | Veneto | Fondo Paviani | FP20 | *Ovis vel capra* | -18.4 | 7.3 | 3.2 | *Tafuri et al. 2018* |
| RBA1–RBA2 | Veneto | Fondo Paviani | FP2 | *Sus domesticus* | -11 | 7.9 | 3.3 | *Tafuri et al. 2018* |
| RBA1–RBA2 | Veneto | Fondo Paviani | FP7 | *Sus domesticus* | -20.2 | 6.9 | 3.2 | *Tafuri et al. 2018* |
| RBA1–RBA2 | Veneto | Fondo Paviani | FP8 | *Sus domesticus* | -11.1 | 9.7 | 3.2 | *Tafuri et al. 2018* |
| RBA1–RBA2 | Veneto | Fondo Paviani | FP13 | *Sus domesticus* | -14 | 7.9 | 3.2 | *Tafuri et al. 2018* |
| RBA2 | Veneto | Fondo Paviani | FP16 | *Sus domesticus* | -20.6 | 4.8 | 3.2 | *Tafuri et al. 2018* |
| RBA2 | Veneto | Fondo Paviani | FP21 | *Sus domesticus* | -11.6 | 9.1 | 3.3 | *Tafuri et al. 2018* |
| RBA2 | Veneto | Fondo Paviani | FP22 | *Sus domesticus* | -16.5 | 8.5 | 3.3 | *Tafuri et al. 2018* |
| RBA2 | Veneto | Fondo Paviani | FP23 | *Sus domesticus* | -13.6 | 8.3 | 3.3 | *Tafuri et al. 2018* |
| MBA | Apulia | Madonna di Loreto | MDLCERVO | *Cervus elaphus* | -20.4 | 7.2 | 3.3 | *Tafuri et al. 2009* |
| MBA | Apulia | Madonna di Loreto | MDLCAPRA | *Ovis vel capra* | -20.4 | 7.1 | 3.3 | *Tafuri et al. 2009* |
| EBA1-MBA2 | Friuli | Mereto | MERETO2 | *Bos taurus* | -20.5 | 4.5 | 3.2 | *Tafuri et al. 2009* |
| EBA1-MBA2 | Friuli | Mereto | ME BOS | *Bos taurus* | -15.1 | 5.1 | 3.2 | *Tafuri et al. 2009, Tafuri et al. 2018* |
| EBA1-MBA3 | Friuli | Mereto | ME_Equus | *Equus sp.* | -20.2 | 4.5 | 3.2 | *Tafuri et al. 2009, Tafuri et al. 2018* |
| RBA2 | Friuli | Gradisca di Codroipo | GRDC q.A3 | *Bos taurus* | -20.4 | 4.2 | 3.2 | *Tafuri et al. 2018* |
| RBA2 | Friuli | Gradisca di Codroipo | GRDC q.DE | *Bos taurus* | -19.3 | 3.4 | 3.6 | *Tafuri et al. 2018* |
| RBA2 | Friuli | Gradisca di Codroipo | GRDC q.E4 | *Bos taurus* | -21.8 | 5.8 | 3.6 | *Tafuri et al. 2018* |
| RBA | Calabria | Punta di Zambrone | SP18 | *Canis familiaris* | -19.7 | 5.3 | 3.2 | *Rumolo et al. 2020* |
| RBA | Calabria | Punta di Zambrone | SP23 | *Canis familiaris* | -16.1 | 6.5 | 2.9 | *Rumolo et al. 2020* |
| RBA | Calabria | Punta di Zambrone | SP24 | *Canis familiaris* | -16.2 | 7.6 | 2.9 | *Rumolo et al. 2020* |
| RBA | Calabria | Punta di Zambrone | SP21 | *Bos taurus* | -17 | 4.7 | 3.4 | *Rumolo et al. 2020* |
| RBA | Calabria | Punta di Zambrone | SP30 | *Bos taurus* | -17.3 | 6 | 3.6 | *Rumolo et al. 2020* |
| RBA | Calabria | Punta di Zambrone | SP31 | *Bos taurus* | -18.2 | 5.2 | 2.8 | *Rumolo et al. 2020* |
| RBA | Calabria | Punta di Zambrone | SP20 | *Sus sp.* | -18.4 | 4.1 | 3.5 | *Rumolo et al. 2020* |
| RBA | Calabria | Punta di Zambrone | SP25 | *Sus sp.* | -19.5 | 4.3 | 2.8 | *Rumolo et al. 2020* |
| RBA | Calabria | Punta di Zambrone | SP26 | *Sus sp.* | -16.5 | 5.8 | 2.9 | *Rumolo et al. 2020* |
| RBA | Calabria | Punta di Zambrone | SP22 | *Cervus elaphus* | -18 | 4.3 | 3.4 | *Rumolo et al. 2020* |
| RBA | Calabria | Punta di Zambrone | SP27 | *Cervus elaphus* | -20.6 | 3 | 3.5 | *Rumolo et al. 2020* |
| RBA | Calabria | Punta di Zambrone | SP29 | *Cervus elaphus* | -19.3 | 4.3 | 3 | *Rumolo et al. 2020* |
| RBA | Calabria | Punta di Zambrone | SP19 | *Ovis vel capra* | -14.9 | 5.7 | 3.4 | *Rumolo et al. 2020* |
| RBA | Calabria | Punta di Zambrone | SP33 | *Ephinephelus* | -18.2 | 5.8 | 2.9 | *Rumolo et al. 2020* |
| CA | Latium | Casetta Mistici | 01N | *Ovis sp.* | -20.2 | 4 | NA | *De Angelis et al. 2015* |
| CA | Latium | Casetta Mistici | 02N | *Bos taurus* | -20.7 | 4.4 | NA | *De Angelis et al. 2015* |
| CA | Latium | Casetta Mistici | 03N | *Ovis sp.* | -20.8 | 4.3 | NA | *De Angelis et al. 2015* |
| CA | Latium | Casetta Mistici | 04N | *Sus sp.* | -20.4 | 4.4 | NA | *De Angelis et al. 2015* |
| CA | Latium | Casetta Mistici | 05N | *Bos taurus* | -20.8 | 4.3 | NA | *De Angelis et al. 2015* |
| CA | Latium | Casetta Mistici | 06N | *Bos taurus* | -21.4 | 4.8 | NA | *De Angelis et al. 2015* |
| CA | Latium | Casetta Mistici | 08N | *Bos taurus* | -20.4 | 4.6 | NA | *De Angelis et al. 2015* |
| CA | Latium | Casetta Mistici | 09N | *Ovis sp.* | -20 | 4.3 | NA | *De Angelis et al. 2015* |
| CA | Latium | Casetta Mistici | 10N | *Ovis sp.* | -18.3 | 4.4 | NA | *De Angelis et al. 2015* |
| CA | Latium | Casetta Mistici | 11N | *Bos taurus* | -20 | 4.8 | NA | *De Angelis et al. 2015* |
| CA | Latium | Casetta Mistici | 12N | *Ovis sp.* | -20.8 | 4.5 | NA | *De Angelis et al. 2015* |
| CA | Latium | Casetta Mistici | 13N | *Sus sp.* | -19.8 | 5.3 | NA | *De Angelis et al. 2015* |
| CA | Latium | Casetta Mistici | 14N | *Sus sp.* | -20.4 | 4.4 | NA | *De Angelis et al. 2015* |
| CA | Latium | Casetta Mistici | 15aN | *Ovis sp.* | -20.4 | 4 | NA | *De Angelis et al. 2015* |
| CA | Latium | Casetta Mistici | 15bN | *Ovis sp.* | -20.4 | 5.1 | NA | *De Angelis et al. 2015* |
| CA | Latium | Casetta Mistici | 15cN | *Ovis sp.* | -19 | 4.1 | NA | *De Angelis et al. 2015* |
| CA | Latium | Casetta Mistici | 16aN | *Bos taurus* | -20.4 | 4.9 | NA | *De Angelis et al. 2015* |
| CA | Latium | Casetta Mistici | 16bN | *Bos taurus* | -20.5 | 4 | NA | *De Angelis et al. 2015* |
| CA | Latium | Casetta Mistici | 17aN | *Bos taurus* | -19.8 | 3.9 | NA | *De Angelis et al. 2015* |
| CA | Latium | Casetta Mistici | 17bN | *Bos taurus* | -20.8 | 5.1 | NA | *De Angelis et al. 2015* |
| CA | Latium | Casetta Mistici | 17cN | *Bos taurus* | -19.2 | 4.3 | NA | *De Angelis et al. 2015* |
| CA | Latium | Casetta Mistici | 18aN | *Ovis sp.* | -20.7 | 4.8 | NA | *De Angelis et al. 2015* |
| CA | Latium | Casetta Mistici | 18bN | *Ovis sp.* | -21 | 3.5 | NA | *De Angelis et al. 2015* |
| CA | Latium | Casetta Mistici | 19aN | *Bos taurus* | -19.5 | 5.5 | NA | *De Angelis et al. 2015* |
| CA | Latium | Casetta Mistici | 19bN | *Bos taurus* | -19.8 | 4.9 | NA | *De Angelis et al. 2015* |
| CA | Latium | Casetta Mistici | 19cN | *Bos taurus* | -19.3 | 5.8 | NA | *De Angelis et al. 2015* |
| CA | Latium | Casetta Mistici | 20aN | *Sus sp.* | -21.3 | 5 | NA | *De Angelis et al. 2015* |
| CA | Latium | Casetta Mistici | 20bN | *Sus sp.* | -20.1 | 4.5 | NA | *De Angelis et al. 2015* |
| CA | Latium | Casetta Mistici | 20cN | *Sus sp.* | -19.7 | 4 | NA | *De Angelis et al. 2015* |
| CA | Latium | Casetta Mistici | 21aN | *Sus sp.* | -19.8 | 4.3 | NA | *De Angelis et al. 2015* |
| CA | Latium | Casetta Mistici | 21bN | *Sus sp.* | -21.3 | 4.8 | NA | *De Angelis et al. 2015* |
| CA | Latium | Casetta Mistici | 21cN | *Sus sp.* | -20.8 | 3.8 | NA | *De Angelis et al. 2015* |
| CA | Latium | Casetta Mistici | 22aN | *Ovis sp.* | -20.8 | 5.2 | NA | *De Angelis et al. 2015* |
| CA | Latium | Casetta Mistici | 22bN | *Ovis sp.* | -20.8 | 5.1 | NA | *De Angelis et al. 2015* |
| CA | Latium | Casetta Mistici | 23aN | *Ovis sp.* | -20.9 | 4.8 | NA | *De Angelis et al. 2015* |
| CA | Latium | Casetta Mistici | 23bN | *Ovis sp.* | -20.3 | 4.5 | NA | *De Angelis et al. 2015* |
| CA | Latium | Casetta Mistici | 24aN | *Ovis sp.* | -20.5 | 5.1 | NA | *De Angelis et al. 2015* |
| CA | Latium | Casetta Mistici | 24bN | *Ovis sp.* | -19.8 | 5.4 | NA | *De Angelis et al. 2015* |
| EBA-MBA | Latium | Pastena | 261 | *Ovis aries vel Capra Hircus* | -20.8 | 5 | 3.3 | *Cortese et al. 2022* |
| EBA-MBA | Latium | Pastena | 23 | *Ovis aries vel Capra Hircus* | -19.9 | 5 | 3.4 | *Cortese et al. 2022* |
| EBA-MBA | Latium | Pastena | 357 | *Ovis aries vel Capra Hircus* | -21.5 | 4.3 | 3.5 | *Cortese et al. 2022* |
| EBA-MBA | Latium | Pastena | 395 | *Ovis aries vel Capra Hircus* | -21.8 | 3.9 | 3.3 | *Cortese et al. 2022* |
| EBA-MBA | Latium | Pastena | 154 | *Sus domesticus* | -21.3 | 4.3 | 3.3 | *Cortese et al. 2022* |
| CA | Marche | Fontenoce Recanati | RE A 106 | Herbivore NA | -20.2 | 4.3 | 3.5 | *De Angelis et al. 2019* |
| CA | Marche | Fontenoce Recanati | RE A 109 | Herbivore NA | -20.7 | 4.3 | 3.3 | *De Angelis et al. 2019* |
| CA | Marche | Fontenoce Recanati | RE A 110 | *Canis familiaris* | -19.3 | 4.9 | 3.3 | *De Angelis et al. 2019* |
| CA | Marche | Fontenoce Recanati | RE A 113 | *Sus scrofa* | -19.7 | 5.6 | 3.3 | *De Angelis et al. 2019* |
| CA | Marche | Fontenoce Recanati | RE A 114 | *Sus scrofa* | -20.9 | 5.9 | 3.3 | *De Angelis et al. 2019* |
| MBA | Tuscany | Grotta Misa | GM_2_F | *Bos taurus* | -19.4 | 6.3 | 3.2 | *Varalli et al. 2016a* |
| MBA | Tuscany | Grotta Misa | GM_1_F | *Bos taurus* | -20.9 | 4.3 | 3.2 | *Varalli et al. 2016a* |
| MBA | Tuscany | Grotta Misa | GM_3_F | Lepus sp. | -20.2 | 3.5 | 3.1 | *Varalli et al. 2016a* |
| MBA | Tuscany | Grotta Misa | GM_4_F | Caprine | -21.7 | 5.6 | 3.2 | *Varalli et al. 2016a* |
| EBA | Tuscany | Grotta dello Scoglietto | SC_070_F | Caprine | -22 | 5.7 | 3.2 | *Varalli et al. 2016a* |
| EBA | Tuscany | Grotta dello Scoglietto | SC_052_F | Caprine | -21.1 | 4.5 | 3.2 | *Varalli et al. 2016a* |
| EBA | Tuscany | Grotta dello Scoglietto | SC_051_F | Caprine | -20.2 | 5.8 | 3.2 | *Varalli et al. 2016a* |
| EBA | Tuscany | Grotta dello Scoglietto | SC_053_F | Caprine | -21.3 | 5.5 | 3.2 | *Varalli et al. 2016a* |
| EBA | Tuscany | Grotta dello Scoglietto | SC_05_F | *Bos taurus* | -18.3 | 6 | 3.2 | *Varalli et al. 2016a* |
| EBA | Tuscany | Grotta dello Scoglietto | SC_014_F | *Bos taurus* | -20.7 | 4.3 | 3.2 | *Varalli et al. 2016a* |
| EBA | Tuscany | Grotta dello Scoglietto | SC_029_F | *Sus sp.* | -20.4 | 5.6 | 3.1 | *Varalli et al. 2016a* |
| EBA | Tuscany | Grotta dello Scoglietto | SC_032_F | *Sus sp.* | -20.5 | 5.4 | 3.1 | *Varalli et al. 2016a* |
| EBA | Tuscany | Grotta dello Scoglietto | SC_031_F | *Sus sp.* | -20.9 | 5.9 | 3.2 | *Varalli et al. 2016a* |
| EBA | Tuscany | Grotta dello Scoglietto | SC_047_F | *Vulpes vulpes* | -20.5 | 7.5 | 3.1 | *Varalli et al. 2016a* |
| EBA | Tuscany | Grotta dello Scoglietto | SC_055_F | *Cervus elaphus* | -20.8 | 4.2 | 3.2 | *Varalli et al. 2016a* |
| EBA | Veneto | Arano di Cellore | bos_1 | *Bos taurus* | -18.6 | 2.9 | 3.2 | *Varalli et al. 2016b* |
| EBA | Veneto | Arano di Cellore | bos_2 | *Bos taurus* | -20.7 | 5.8 | 3.3 | *Varalli et al. 2016b* |
| EBA | Veneto | Arano di Cellore | bos_4 | *Bos taurus* | -18.2 | 4.2 | 3.3 | *Varalli et al. 2016b* |
| EBA | Veneto | Arano di Cellore | cervus_1 | *Cervus elaphus* | -17.4 | 5.3 | 3.2 | *Varalli et al. 2016b* |
| EBA | Veneto | Arano di Cellore | cervus_2 | *Cervus elaphus* | -20 | 3.2 | 3.2 | *Varalli et al. 2016b* |
| EBA | Veneto | Arano di Cellore | sus_1 | *Sus sp.* | -20.8 | 4.7 | 3.3 | *Varalli et al. 2016b* |
| EBA | Veneto | Arano di Cellore | sus_2 | *Sus sp.* | -20.4 | 4.6 | 3.2 | *Varalli et al. 2016b* |
| EBA | Veneto | Arano di Cellore | sus_3 | *Sus sp.* | -20.6 | 4.7 | 3.3 | *Varalli et al. 2016b* |
| EBA | Veneto | Arano di Cellore | sus_4 | *Sus sp.* | -20.3 | 6.1 | 3.2 | *Varalli et al. 2016b* |
| EBA | Veneto | Arano di Cellore | sus_5 | *Sus sp.* | -20.2 | 3.6 | 3.2 | *Varalli et al. 2016b* |
| EBA | Veneto | Arano di Cellore | c_o_1 | *Ovis vel capra* | -19.2 | 4.9 | 3.2 | *Varalli et al. 2016b* |
| EBA | Veneto | Arano di Cellore | c_o_2 | *Ovis vel capra* | -19.6 | 4.8 | 3.2 | *Varalli et al. 2016b* |
| EBA | Veneto | Arano di Cellore | c_o_3 | *Ovis vel capra* | -20.7 | 2.8 | 3.2 | *Varalli et al. 2016b* |
| BA | Basilicata | Murgia Timone | MT01 | *Ovis sp.* | -20.7 | 5.6 | 3.3 | *Arena et al. 2020* |
| BA | Basilicata | Murgia Timone | MT02 | *Canis familiaris* | -19.1 | 8 | 3.2 | *Arena et al. 2020* |
| BA | Basilicata | Murgia Timone | MT03 | *Bos taurus* | -19.2 | 7.3 | 3.3 | *Arena et al. 2020* |
| BA | Basilicata | Murgia Timone | MT04 | *Sus sp.* | -20.4 | 7.7 | 3.2 | *Arena et al. 2020* |
| BA | Basilicata | Murgia Timone | MT05 | *Bos taurus* | -19.1 | 7.4 | 3.2 | *Arena et al. 2020* |
| CA | Calabria | Grotta di Donna Marsilia | GdMR02 | *Sus sp.* | -21.4 | 4.1 | 3.2 | *Arena et al. 2020* |
| CA | Calabria | Grotta di Donna Marsilia | GdMR03 | *Sus sp.* | -19.7 | 8.5 | 3.3 | *Arena et al. 2020* |
| CA | Calabria | Grotta di Donna Marsilia | GdMR04 | *Sus sp.* | -20.7 | 3 | 3.2 | *Arena et al. 2020* |
| MBA | Latium | Regina Margherita |  | *Ovis sp.* | -21.3 | 3.7 | 3.3 | *Skeates et al. 2021* |
| MBA | Latium | Regina Margherita |  | *Ovis sp.* | -19.6 | 4.5 | 3.3 | *Skeates et al. 2021* |
| MBA | Latium | Regina Margherita |  | *Ovis sp.* | -21 | 4.5 | 3.3 | *Skeates et al. 2021* |
| MBA | Latium | Regina Margherita |  | *Ovis sp.* | -21.2 | 4.4 | 3.4 | *Skeates et al. 2021* |
| MBA | Latium | Regina Margherita |  | *Ovis sp.* | -20.8 | 3 | 3.2 | *Skeates et al. 2021* |
| MBA | Latium | Regina Margherita |  | *Ovis sp.* | -20.8 | 4.3 | 3.3 | *Skeates et al. 2021* |
| MBA | Latium | Regina Margherita |  | *Bos taurus* | -21.6 | 4.5 | 3.4 | *Skeates et al. 2021* |
| MBA | Latium | Regina Margherita |  | *Bos taurus* | -19.8 | 4.3 | 3.4 | *Skeates et al. 2021* |
| MBA | Latium | Regina Margherita |  | *Cervus elaphus* | -20.9 | 4.3 | 3.4 | *Skeates et al. 2021* |
| MBA | Latium | Regina Margherita |  | *Vulpes vulpes* | -19 | 7.7 | 3.3 | *Skeates et al. 2021* |
| MBA | Latium | Regina Margherita |  | *Vulpes vulpes* | -19 | 8 | 3.3 | *Skeates et al. 2021* |
| MBA | Latium | Regina Margherita |  | *Canis familiaris* | -19.6 | 6.7 | 3.3 | *Skeates et al. 2021* |
| MBA | Latium | Regina Margherita |  | *Canis familiaris* | -19.4 | 6.7 | 3.3 | *Skeates et al. 2021* |
| EBA-MBA | Lombardy | Ballabio | BA_A1 | *Ovis sp.* | -20.8 | 3.4 | 3.3 | *Masotti et al. 2019* |
| EBA-MBA | Lombardy | Ballabio | BA_A2 | *Sus sp.* | -20.7 | 4.3 | 3.2 | *Masotti et al. 2019* |
| EBA-MBA | Lombardy | Ballabio | BA_A3 | *Bos taurus* | -20.3 | 4.7 | 3.2 | *Masotti et al. 2019* |

Table S5: **Stable carbon and nitrogen isotope ratios in animal bone collagen from CA and BA samples from Italy.** NA=Not Available; BA=Bronze Age; CA=Copper Age; MBA=Middle Bronze Age; EBA1–EBA2=Early Bronze Age sub-phase 1/Early Bronze Age sub-phase 2; RBA1–RBA2=Recent Bronze Age 1/ Recent Bronze Age 2; RBA2=Recent Bronze Age 2; EBA1-MBA2=Early Bronze Age sub-phase 1/Middle Bronze Age sub-phase 2; EBA1-MBA3=Early Bronze Age sub-phase 1/Middle Bronze Age sub-phase 3; RBA=Recent Bronze Age; EBA-MBA=Early Bronze Age/Middle Bronze Age; EBA=Early Bronze Age.

References table S5:

Bernardini S, Coppa A, Moggi-Cecchi J, Barbaro CC, Alhaique F, Tagliacozzo A, et al. Social Dynamics and Resource Management Strategies in Copper Age Italy: Insights from Archaeological and Isotopic Data. Environmental Archaeology. 2021; 1–23. doi:10.1080/14614103.2021.1891812;

Tafuri MA, Rottoli M, Cupitò M, Pulcini ML, Tasca G, Carrara N, et al. Estimating C4 plant consumption in Bronze Age Northeastern Italy through stable carbon and nitrogen isotopes in bone collagen. International Journal of Osteoarchaeology. 2018;28: 131–142. doi:10.1002/oa.2639

Rumolo A, Forstenpointner G, Rumolo P, Jung R. Palaeodiet reconstruction inferred by stable isotopes analysis of faunal and human remains at Bronze Age Punta di Zambrone (Calabria, Italy). International Journal of Osteoarchaeology. 2020;30: 90–98. doi:10.1002/oa.2836

De Angelis F, Di Giannantonio S, Scorrano G, Catalano P, Rickards O. Approccio integrato ai metodi di sussistenza delle comunità eneolitiche di Via Casetta Mistici e Osteria del Curato-Via Cinquefrondi. Atti RIUNIONE DI LAVORO PRIN 2010-2011. ISBN9788865079065.

Cortese F, De Angelis F, Achino KF, Bontempo L, di Cicco MR, Gatta M, et al. Isotopic reconstruction of the subsistence strategy for a Central Italian Bronze Age community (Pastena cave, 2nd millennium BCE). Archaeological and Anthropological Sciences. 2022;14: 201. doi:10.1007/s12520-022-01673-5

De Angelis F, Scorrano G, Martínez-Labarga C, Giustini F, Brilli M, Pacciani E, et al. Eneolithic subsistence economy in Central Italy: first dietary reconstructions through stable isotopes. Archaeological and Anthropological Sciences. 2019;11: 4171–4186. doi:10.1007/s12520-019-00789-5

Varalli A, Moggi-Cecchi J, Moroni A, Goude G. Dietary Variability During Bronze Age in Central Italy: First Results. International Journal of Osteoarchaeology. 2016a;26: 431–446. doi:10.1002/oa.2434

Varalli A, Moggi-Cecchi J, Dori I, Boccone S, Bortoluzzi S, Salzani P, et al. Dietary continuity vs. discontinuity in Bronze Age Italy. The isotopic evidence from Arano di Cellore (Illasi, Verona, Italy). Journal of Archaeological Science: Reports. 2016b;7: 104–113. doi:10.1016/j.jasrep.2016.03.047

Arena F, Gualdi-Russo E, Olsen J, Philippsen B, Mannino MA. New data on agro-pastoral diets in southern Italy from the Neolithic to the Bronze Age. Archaeological and Anthropological Sciences. 2020;12: 245–245. doi:10.1007/s12520-020-01209-9

Skeates R, Beckett J, Mancini D, Cavazzuti C, Silvestri L, Hamilton WD, et al. Rethinking Collective Burial in Mediterranean Caves: Middle Bronze Age Grotta Regina Margherita, Central Italy. Journal of Field Archaeology. 2021;46: 382–398. doi:10.1080/00934690.2021.1917137

Masotti S, Varalli A, Goude G, Moggi-Cecchi J, Gualdi-Russo E. A combined analysis of dietary habits in the Bronze Age site of Ballabio (northern Italy). Archaeological and Anthropological Sciences. 2019;11: 1029–1047. doi:10.1007/s12520-017-0588-0

Table S6: **Mann-Whitney U test results for the comparison of CA human samples.** Lower Triangle: U values; Upper Triangle: p-values. Not significant p-values are in bold. ^1^ This study.^2^ De Angelis, F. et al. Eneolithic subsistence economy in Central Italy: first dietary reconstructions through stable isotopes. Archaeol. Anthropol. Sci. 11, 4171–4186 (2019).^3^ De Angelis, F., Di Giannantonio, S., Scorrano, G., Catalano, P. & Rickards, O. An integrated approach to subsistence of the Eneolithic communities of Via Casetta Mistici and Osteria del Curato - Via Cinquefrondi. Biol. Cult. Herit. Cent.-South. Ital. Popul. 30 Thousand Years (2015).^4^ Bernardini, S. et al. Social Dynamics and Resource Management Strategies in Copper Age Italy: Insights from Archaeological and Isotopic Data. Environ. Archaeol. 0, 1–23 (2021).


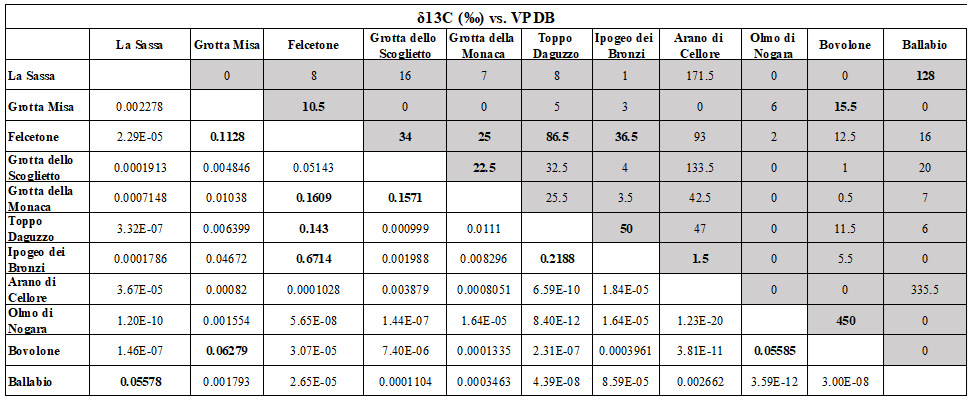

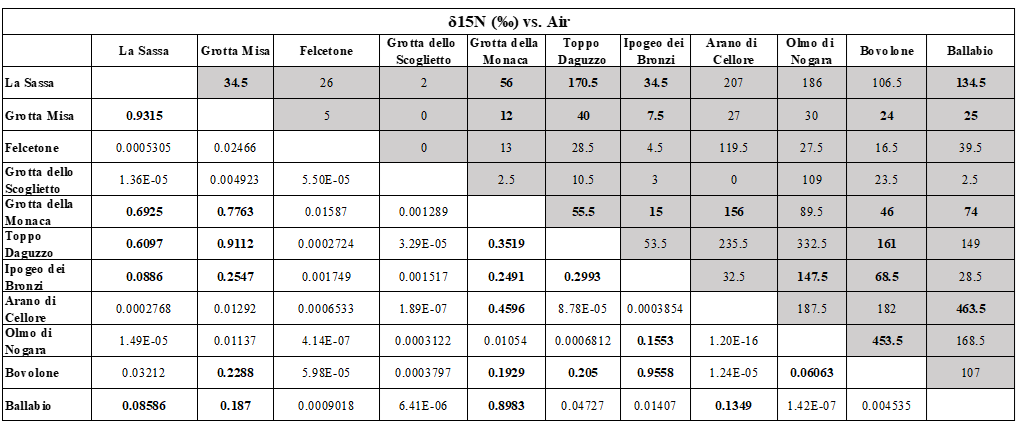


### Table S7: Coupled Mann-Whitney test between Bronze Age Italian sites for δ13C and δ15N values. Significant p values are reported in Bold.

## 5 Mobility patterns


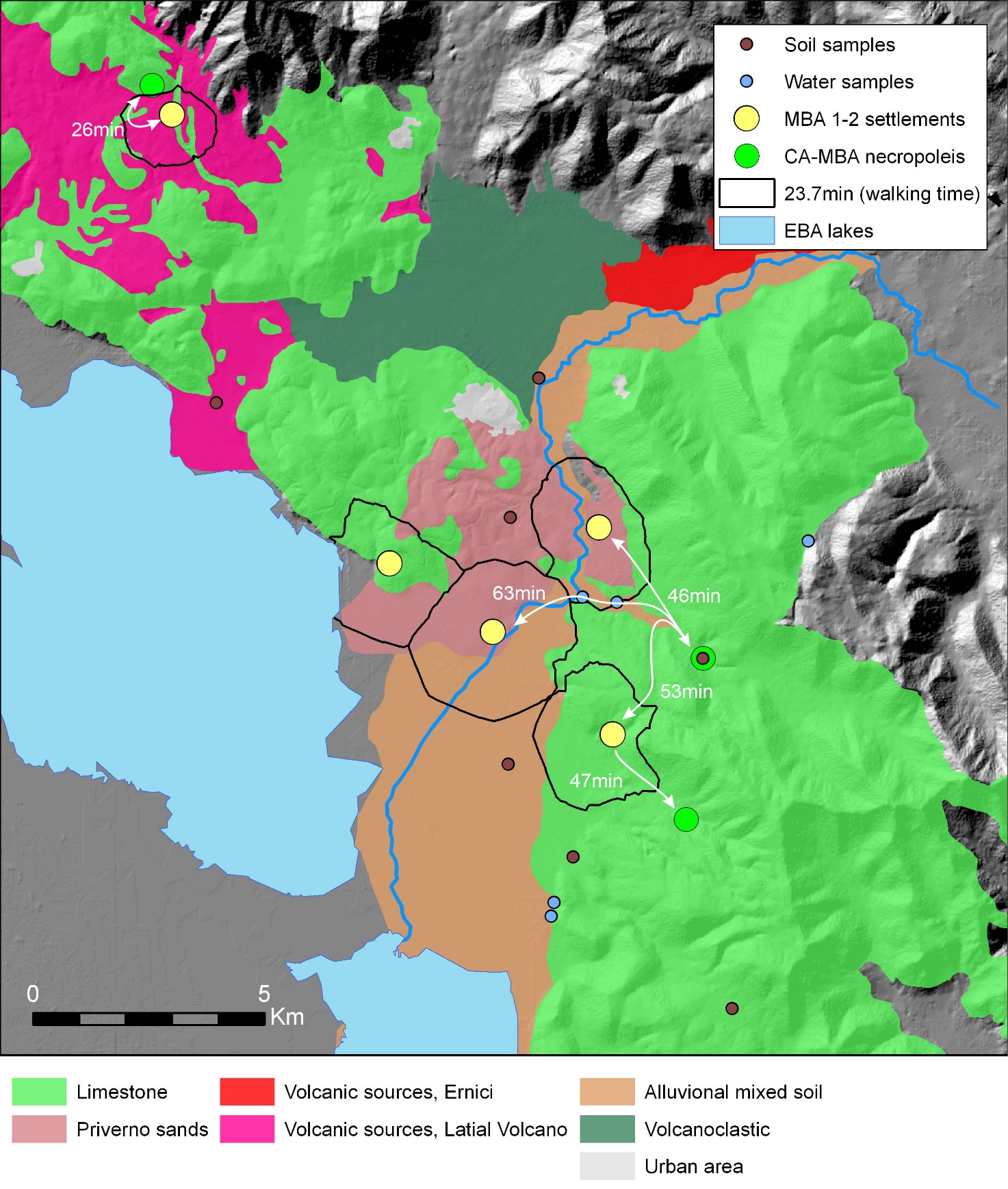


Fig. S6: **the reconstructed territories for the MBA sites and the walking time distance from the nearby (known) necropoleis.** The funerary use of the Pistocchino cave (the southernmost necropolis) is hypothetic. Territory reconstructions from Alessandri 2013^36^ and lake reconstruction from Van Gorp et al. 2020^19^, background DEM from TINITALY/01^37^

## 6 Radiocarbon dates

| **Lab code** | **Sample code** | **Context** | **Sample** | **14C age** | **STD** | **Calibrated Age (OxCal4.3, IntCal13, 95.4%)** | **Bibliography** |
| --- | --- | --- | --- | --- | --- | --- | --- |
| LTL19066A | LS 2993 | Room 1, SU 97 | Human femur | 4409 | 45 | 3327 - 2911 calBC | 38 |
| LTL19064 | LS 605 | Room 1, SU 55 | Human femur | 3722 | 40 | 2278 - 1980 calBC | 38 |
| LTL19065A | LS 873 | Room 1, SU 78 | Human femur | 3451 | 45 | 1888 - 1646 calBC | 38 |
| LTL17395A | LS 430 | Room 1, SU 9 | Sus sp., right ulna | 3148 | 45 | 1506 - 1293 calBC | 38 |
| LTL18165 | LS 1040 | Room 1, SU 26 | Sus sp., radius | 3112 | 40 | 1492 - 1266 calBC | 38 |
| GrA64828 | LS 418 | Room 2, SU 19 | Human femur | 4000 | 35 | 2619 - 2462 calBC | 38 |
| UBA 41603 | LS 200+155 | Room 2, SU 19 | Human, petrous bone | 4091 | 29 | 2860 - 2500 calBC | 39 |
| UBA 41605 | LS 346 | Room 2, SU 19 | Human tooth | 4097 | 39 | 2868 - 2497 calBC | 39 |
| UBA-41606 | LS 150 | Room 2, SU 19 | Human tooth | 4073 | 37 | 2859 - 2488 calBC | 39 |
| LTL18166 | LS 1047 | Room 2, SU 31 | Ovis sp., right humerus | 3205 | 45 | 1611 - 1406 calBC | 38 |
| LTL17393A | LS 425 | Room 3, SU 7 | Sus sp., left radius | 3101 | 45 | 1492 - 1233 calBC | 38 |
| LTL18164 | LS 1014 | Room 4, SU 25 | Indeterminate, vertebra | 4271 | 45 | 3019 - 2701 calBC | 38 |
| LTL20395A | LS 2176 | Area RA, SU 134 | Human femur | 3165 | 40 | 1519 - 1306 calBC | 38 |

Table S8**: radiocarbon dates for the Copper age and the Bronze age at La Sassa**

## 7 References for chapters 1-6

1. Sevink, J., Remmelzwaal, A. & Spaargaren, O. C. *The soils of southern Lazio and adjacent Campania*. (Universiteit van Amsterdam, 1984).

2. Cosentino, D., Cipollari, P., Marsili, P. & Scrocca, D. Geology of the central Apennines: a regional review. *J. Virtual Explor.* **36**, (2010).

3. Peccerillo, A. The Ernici-Roccamonfina Province BT - Cenozoic Volcanism in the Tyrrhenian Sea Region. in (ed. Peccerillo, A.) 125–143 (Springer International Publishing, 2017). doi:10.1007/978-3-319-42491-0_5

4. Marra, F. *et al.* The Volsci Volcanic Field (central Italy): eruptive history, magma system and implications on continental subduction processes. *Int. J. Earth Sci.* (2021). doi:10.1007/s00531-021-01981-6

5. Arnoldus-Huyzendveld, A., Ketting, A. & Sevink, J. *Indagine comparativa pedogenetica sul Tardo Quaternario nel Lazio meridionale. Progetto Sicurezza degli Impianti a Fronte di Eventi Naturali.* (1985).

6. Arnoldus-Huyzendveld, A., Perotto, C. & Sarandrea, P. *I suoli della provincia di Latina : carta, database e applicazioni*. (Gangemi, 2009).

7. Spaargaren, O. C. *Weathering and soil formation in a limestone area near Pastena (Fr., Italy)*. (1979).

8. Moresi, M. & Mongelli, G. The relation between the terra rossa and the carbonate-free residue of the underlying limestones and dolostones in Apulia, Italy. *Clay Miner.* **23**, 439–446 (1988).

9. Mirabella, A., Costantini, E. A. C. & Carnicelli, S. Genesis of a polycyclic Terra Rossa (Chromic Cambisol on Rhodic Nitisol) at the Poggio del Comune in Central Italy. *Zeitschrift für Pflanzenernährung und Bodenkd.* **155**, 407–413 (1992).

10. Yaalon, D. H. Soils in the Mediterranean region: what makes them different? *CATENA* **28**, 157–169 (1997).

11. Vingiani, S., Di Iorio, E., Colombo, C. & Terribile, F. Integrated study of Red Mediterranean soils from Southern Italy. *CATENA* **168**, 129–140 (2018).

12. Durn, G., Ottner, F. & Slovenec, D. Mineralogical and geochemical indicators of the polygenetic nature of terra rossa in Istria, Croatia. *Geoderma* **91**, 125–150 (1999).

13. Priori, S. *et al.* Pedostratigraphy of Terra Rossa and Quaternary geological evolution of a lacustrine limestone plateau in central Italy. *J. Plant Nutr. Soil Sci.* **171**, 509–523 (2008).

14. Stuut, J.-B., Smalley, I. & O’Hara-Dhand, K. Aeolian dust in Europe: African sources and European deposits. *Quat. Int.* **198**, 234–245 (2009).

15. Remmelzwaal, A. *Soil Genesis and Quaternary Landscape Development in the Tyrrhenian Coastal Area of South-Central Italy*. (1978).

16. Funiciello, R., Giordano, G. & Mattei, M. *Geological Map of Roma Municipality, Scale 1:50.000*. (SELCA, 2008).

17. *The Colli Albani Volcano*. (The Geological Society, 2010).

18. van Gorp, W. & Sevink, J. Distal deposits of the Avellino eruption as a marker for the detailed reconstruction of the Early Bronze Age depositional environment in the Agro Pontino and Fondi Basin (Lazio, Italy). *Quat. Int.* **499B**, 245–257 (2019).

19. van Gorp, W., Sevink, J. & Van Leusen, M. Post-depositional subsidence of the Avellino tephra marker bed in the Pontine plain (Lazio, Italy): Implications for Early Bronze Age palaeogeographical, water level and relative sea level reconstruction. *CATENA* **194**, 104770 (2020).

20. Van Joolen, E. *Archaeological land evaluation A reconstruction of the suitability of ancient landscapes for various land uses in Italy focused on the first millennium BC*. (2003).

21. Sponheimer, M. & Lee-Thorp, J. A. Alteration of Enamel Carbonate Environments during Fossilization. *J. Archaeol. Sci.* **26**, 143–150 (1999).

22. Pucéat, E., Reynard, B. & Lécuyer, C. Can crystallinity be used to determine the degree of chemical alteration of biogenic apatites? *Chem. Geol.* **205**, 83–97 (2004).

23. Beasley, M. M., Bartelink, E. J., Taylor, L. & Miller, R. M. Comparison of transmission FTIR, ATR, and DRIFT spectra: implications for assessment of bone bioapatite diagenesis. *J. Archaeol. Sci.* **46**, 16–22 (2014).

24. Wopenka, B. & Pasteris, J. D. A mineralogical perspective on the apatite in bone. *Mater. Sci. Eng. C* **25**, 131–143 (2005).

25. Rey, C., Renugopalakrishman, V., Collins, B. & Glimcher, M. J. Fourier transform infrared spectroscopic study of the carbonate ions in bone mineral during aging. *Calcif. Tissue Int.* **49**, 251–258 (1991).

26. Madupalli

27. Piga, G. *et al.* A case of semi-combusted pregnant female in the Phoenician-Punic necropolis of Monte Sirai (Carbonia, Sardinia, Italy). *HOMO* **67**, 50–64 (2016).

28. Paba, R., Thompson, T. J. U., Fanti, L. & Lugliè, C. Rising from the ashes: A multi-technique analytical approach to determine cremation. A case study from a Middle Neolithic burial in Sardinia (Italy). *J. Archaeol. Sci. Reports* **36**, 102855 (2021).

29. Wright, L. E. & Schwarcz, H. P. Infrared and Isotopic Evidence for Diagenesis of Bone Apatite at Dos Pilas, Guatemala: Palaeodietary Implications. *J. Archaeol. Sci.* **23**, 933–944 (1996).

30. Morris, M. D. & Mandair, G. S. Raman assessment of bone quality. *Clin. Orthop. Relat. Res.* **469**, 2160–2169 (2011).

31. Trueman, C. N. G., Behrensmeyer, A. K., Tuross, N. & Weiner, S. Mineralogical and compositional changes in bones exposed on soil surfaces in Amboseli National Park, Kenya: diagenetic mechanisms and the role of sediment pore fluids. *J. Archaeol. Sci.* **31**, 721–739 (2004).

32. Madejova

33. Trueman, C. N., Privat, K. & Field, J. Why do crystallinity values fail to predict the extent of diagenetic alteration of bone mineral? *Palaeogeogr. Palaeoclimatol. Palaeoecol.* **266**, 160–167 (2008).

34. Fernandes, R. and Millard, A.R. and Brabec, M. and Nadeau, M.-J. and Grootes, P. Food Reconstruction Using Isotopic Transferred Signals (FRUITS) : a Bayesian model for diet reconstruction., *PLoS ONE*, **9** (2). e87436. (2014).

35. Cortese F, Angelis FD, Achino KF, Bontempo L, Cicco MRD, Gatta M, et al. Isotopic reconstruction of the subsistence strategy for a Central Italian Bronze Age community (Pastena cave, 2nd millennium BCE). *Archaeol Anthropol Sci* **14**, 201 (2022).

36. Alessandri, L. *Latium Vetus in the Bronze Age and Early Iron Age / Il Latium Vetus nell’età del Bronzo e nella prima età del Ferro*. (BAR International Series, 2565, 2013).

37. Tarquini, S. *et al.* TINITALY/01: a new Triangular Irregular Network of Italy. *Ann. Geophys.* **50**, 407–425 (2007).

38. Alessandri, L., Cardello, G.L., Attema, P.A.J., Baiocchi, V., De Angelis, F., Del Pizzo, S., Di Ciaccio, F., Fiorillo, A., Gatta, M., Monti, F., Onori, M., Rolfo, M.F., Romboni, M., Sottili, G., Troisi, S. Reconstructing the Late Pleistocene – Anthropocene interaction between the neotectonic and archaeological landscape evolution in the Apennines (La Sassa cave, Italy). *Quaternary Science Reviews*. **265**, 107067–107067 (2021). https://doi.org/10.1016/j.quascirev.2021.107067

39. Saupe, T., Montinaro, F., Scaggion, C., Carrara, N., Kivisild, T., D’Atanasio, E., Hui, R., Solnik, A., Lebrasseur, O., Larson, G., Alessandri, L., Arienzo, I., De Angelis, F., Rolfo, M.F., Skeates, R., Silvestri, L., Beckett, J., Talamo, S., Dolfini, A., Miari, M., Metspalu, M., Benazzi, S., Capelli, C., Pagani, L., Scheib, C.L. Ancient genomes reveal structural shifts after the arrival of Steppe-related ancestry in the Italian Peninsula. *Current Biology*. https://doi.org/10.1016/j.cub.2021.04.022 (2021).

## 8 Potsherds from the sounding SP


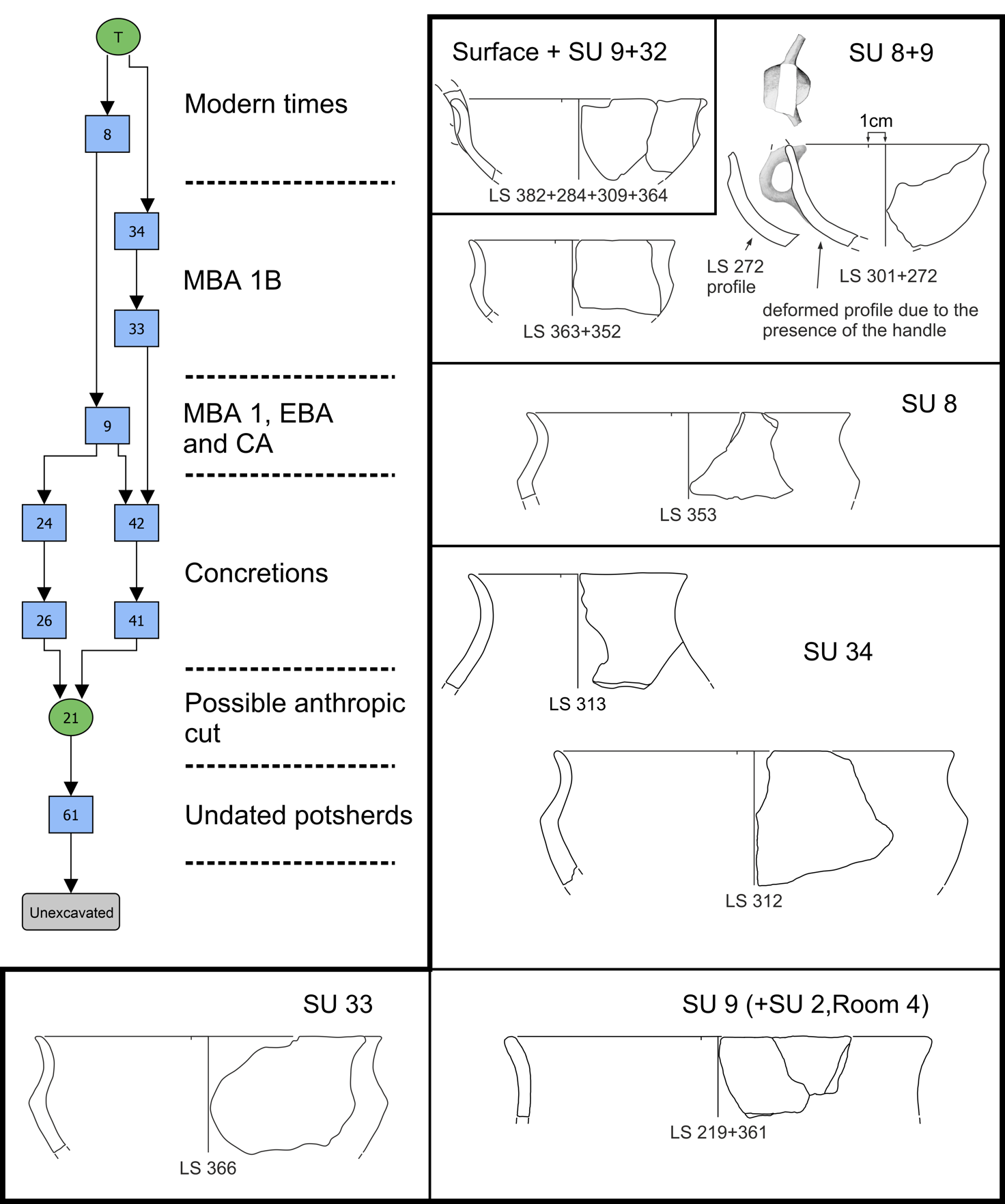


Fig. S7: **potsherds from the sounding SP in the La Sassa cave.** On the left, the matrix of the sounding. SU, Stratigraphic Unit; LS, La Sassa (the prefix of the potsherds number). Individual scale, always 1 cm.


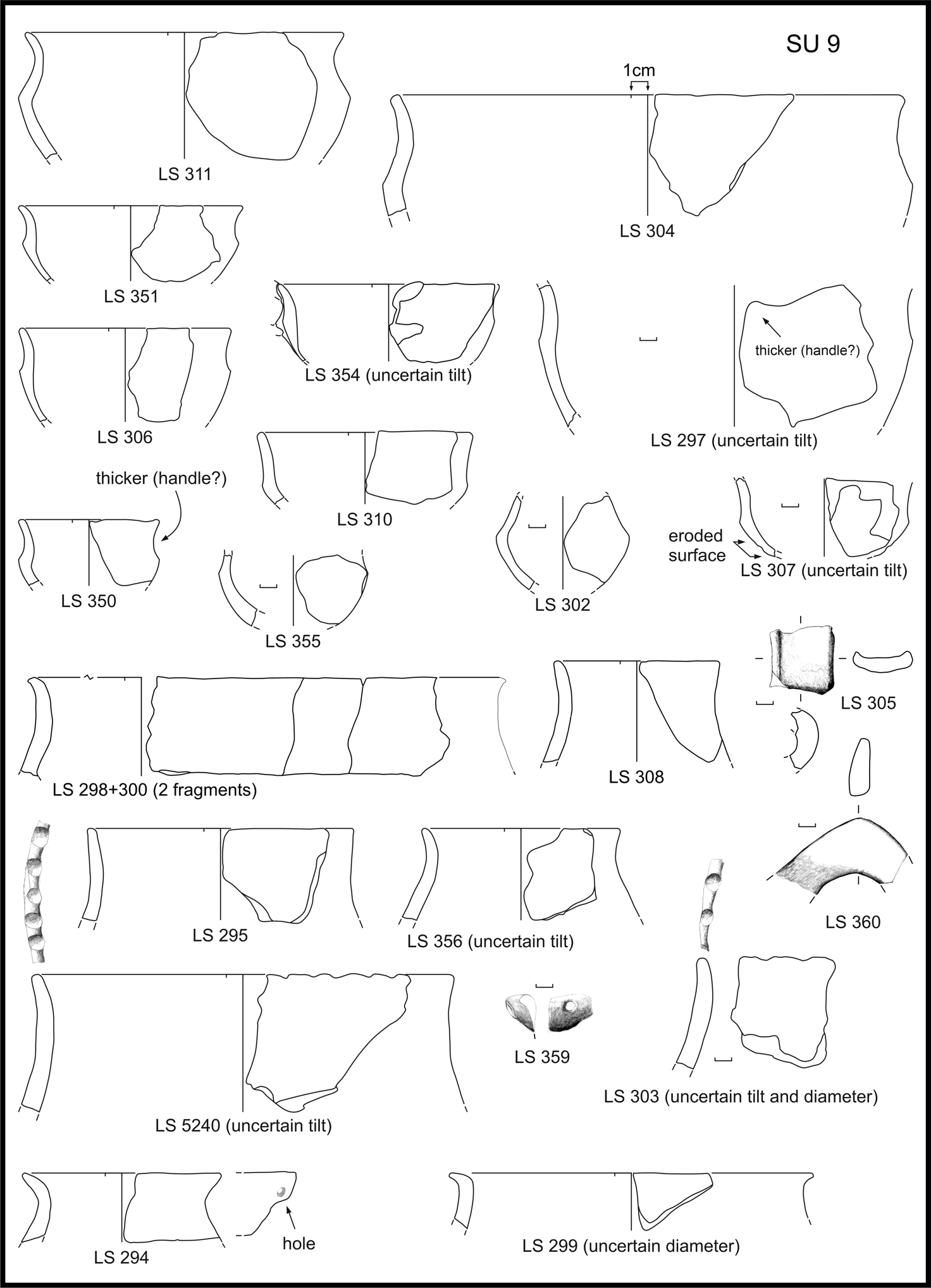


Fig. S8: **potsherds from the Stratigraphic Unit 9, in the sounding SP, La Sassa cave.** LS, La Sassa (the prefix of the potsherds number). Individual scale, always 1 cm.

| **Potsherds number** | **Room** | **Context** | **Parallels** | **Chronology** |
| --- | --- | --- | --- | --- |
| LS 382 (sur) + 284 (US 32) + 309 (US 9) + 364 (US 9) | 1 | Sector SP, Surface, USs 1, 9 and 32 | **Camposauro**, (Talamo 1996, n. 3) | EBA |
| LS 353 | 1 | Sector SP, US 8 | **Spiagge Sant’Agostino** (Alessandri 2007, fig. 3.133, 3) | MBA 1/2 |
| LS 301 (US 9) + 272 (US 8) | 1 | Sector SP, USs 8 and 9 | **Grotta Regina Margherita** (Guidi 1981, fig. 5, 1) | BM 1 (Cocchi Genick 1995, type 229) |
| LS 363 (US 9) + 352 (US 8) | 1 | Sector SP, USs 8 and 9 | **San Paolo Belsito, Montesano**, above AV (Albore Livadie et al. 2007, figs 1, 4) | MBA 1 |
|  |  |  | **Boscoreale** (Albore Livadie 2007, fig. 1,5) | EBA 1 |
|  |  |  | **Oliva Torricella** (Soriano and Albore Livadie, fig. 3, first column from the left, third row) | EBA |
| LS 294 | 1 | Sector SP, US 9 | **Grotta di San Giuseppe** (Cremonesi 2001, fig. 6, 6) | CA |
| LS 295 | 1 | Sector SP, US 9 | **Trinità**, Piano di Sorrento (Esposito 1990, tav. 22, 227319) | CA |
| LS 297 | 1 | Sector SP, US 9 | **Grotta Cardini**, Strato Medio (Bernabò Brea 1985, fig. 7b) | MBA 1/2A (Damiani 1995) |
| LS 298 + 300 | 1 | Sector SP, US 9 | **Riparo del Lauro**, Candalla (Cocchi Genick 1987, fig. 19, 1) | MBA 1B (Cocchi Genick 2001, type 320) |
| LS 299 | 1 | Sector SP, US 9 | **Lago di Mezzano,** (Petitti and Mitchell 1993, tav. 9, 3) | EBA 2 (Cocchi Genick 1998, unicum u.1d.140) |
| LS 302 | 1 | Sector SP, US 9 | see LS 355 |  |
| LS 303 | 1 | Sector SP, US 9 | **Spigolizzi** (Ingravallo, Piccinno 1985, tav. 37, 3) | MBA1 (Damiani 1995, type 355) |
| LS 304 | 1 | Sector SP, US 9 | **Caprolace** (Alessandri 2013, fig. 207.2, 12 and 17) | MBA 1 to RBA |
|  |  |  |  |  |
|  |  |  | **Grotta Cardini**, Strato Medio, Scavo 1966, Taglio 9 (Cavalier 1989, fig. 51l) | MBA 1/2A (Damiani 1995) |
| LS 305 | 1 | Sector SP, US 9 | Vertical band handle on the rim, typical of both EBA and MBA | EBA and MBA |
| LS 306 | 1 | Sector SP, US 9 | **Grotta Regina Margherita** (Guidi 1981, fig. 5,1) | MBA 1 (Cocchi Genick 1995, type 229) |
| LS 307 | 1 | Sector SP, US 9 | See LS 310 |  |
| LS 308 | 1 | Sector SP, US 9 | **Gaudo,** Tomba “e” (Aurino 2014, fig. 5, A11) | CA |
|  |  |  | **Osteria del Curato - Via Cinquefrondi**, grave 29 (Anzidei at al. 2007, fig. 3, 6) | CA |
| LS 310 | 1 | Sector SP, US 9 | **Saviano, Masseria Tufano** (Albore Livadie 1999, figs 4, C2) | EBA |
| LS 311 | 1 | Sector SP, US 9 | **Riparo Grande di Camaiore**, US 4 (Cocchi Genick 1992, fig. 1,2 and 4) | MBA 1B (Cocchi 2002, p. 49) |
| LS 350 | 1 | Sector SP, US 9 | **Monte Fellino, Calcara Pagliara** (Albore Livadie 1999, figs 7, 7) | EBA |
|  |  |  | **S. Maria in Belverde**, strato 3 (Cuda and Sarti 1996, fig. 1,2) | EBA |
| LS 351 | 1 | Sector SP, US 9 | **Spiagge Sant’Agostino** (Guidi 1980, fig. 1, 5) | MBA 1/2 (Cocchi Genick 1995 type 224B) |
| LS 354 | 1 | Sector SP, US 9 | See LS 351 |  |
| LS 355 | 1 | Sector SP, US 9 | **Oliva Torricella** (Soriano and Albore Livadie 2017, fig. 2, second column from the left, last row) | EBA |
| LS 356 | 1 | Sector SP, US 9 | **Broglio di Trebisacce,** str. 4W (Bergonzi et alii, tav. 2,1) | MBA 1-3 (Damiani 1995, type 430B) |
| LS 359 | 1 | Sector SP, US 9 | **Torre Crognola** (Pennacchioni 1977, tav. 22, 48) | CA |
|  |  |  | **Le Cerquete-Fianello** (Carboni 2002, fig. 48, 3) | CA |
| LS 360 | 1 | Sector SP, US 9 | **Lago di Mezzano**, (Franco 1982, tav. XLII, M2-41) | BM 2/3 (Cocchi Genick 2001, type 29) |
| LS 5240 | 1 | Sector SP, US 9 | **Spigolizzi** (Ingravallo, Piccinno 1985, tav. 37, 3) | MBA1 (Damiani 1995) |
| LS 366 | 1 | Sector SP, US 33 | See LS 311 |  |
| LS 312 | 1 | Sector SP, US 34 | See LS 311 |  |
| LS 313 | 1 | Sector SP, US 34 | **S. Marco di Metaponto** (Bianco 1978, fig. 4,1) | MBA 1 (Damiani 1995 type 355) |
|  |  |  | **Grotta di San Giuseppe** (Cremonesi 2001, fig. 6, 4) | CA |
| LS 219 + 361 | 1, 4 | Sector SP, US 9; Room 4, US 2 | **Grotta Cardini**, Strato Medio (Bernabò Brea 1985, fig. 7,b) | MBA 1/2 (Cocchi Genick 1995 type 121) |

Table S9: **parallels and chronology for the ceramics from sounding SP**

**References for parallels (table S9)**

Albore Livadie, C. 1999, Territorio ed insediamenti nell’Agro Nolano durante il Bronzo antico (facies di Palma Campania): nota preliminare, in Albore Livadie, C (ed), *L’eruzione vesuviana delle “Pomici di Avellino” e la facies di Palma Campania (Bronzo Antico),* Bari, pp. 203–246.

Albore Livadie, C., Vecchio, G., Castaldo, N. 2007, L’età del Bronzo a San Paolo Belsito (Nola – Napoli), in *Strategie di insediamento fra Lazio e Campania in età preistorica e protostorica*, proceedings of the XL Riunione Scientifica dell’Istituto Italiano di Preistoria e Protostoria, pp. 869–872.

Alessandri, L. 2007, *L’occupazione costiera protostorica del Lazio centromeridionale*. BAR International Series, 1592, Oxford 2007.

Alessandri, L. 2013, *Latium Vetus in the Bronze Age and Early Iron Age / Il Latium Vetus nell’età del Bronzo e nella prima età del Ferro*. BAR International Series, 2565, Oxford 2013.

Anzidei, A.P., Carboni, G., Castagna, M.A., Celant, A., Cianca, M., Egidi, R., Favorito, S., Funiciello, R., Giordano, G., Malvone, M., Tagliacozzo, A. 2007, L’abitato eneolitico di Osteria del Curato - Via Cinquefrondi: nuovi dati sulle facies archeologiche di Laterza e Ortucchio nel territorio di Roma, in *Strategie Di Insediamento Fra Lazio e Campania in Età Preistorica e Protostorica*, proceedings of the XL Riunione Scientifica dell’Istituto Italiano di Preistoria e Protostoria, pp. 477–508.

Aurino, P. 2014, La necropoli eneolitica del Gaudo (Paestum) tra scoperta e riscoperte, in Guidi, A. (ed), *150 Anni Di Preistoria e Protostoria in Italia*, Firenze 2014, pp. 437–444.

Bergonzi, G., Cardarelli, A., Vagnetti, L., Peroni, R. 1982, Broglio di Trebisacce, in Guzzo, P.G., Peroni, R., Bergonzi, G., Cardarelli, A., Vagnetti (eds), *Ricerche sulla protostoria della Sibaritide,* 1, pp. 51–93.

Bernabò Brea, L. 1985, *Gli Eoli e l’inizio dell’età del Bronzo nelle isole Eolie e nell’Italia meridionale. Archeologia e leggende*, Napoli 1985.

Bianco S. 1978, I materiali provenienti dal villaggio dell'Età del Bronzo di S. Marco presso Metaponto (Matera), in proceedings of the XX Riunione Scientifica dell’Istituto Italiano di Preistoria e Protostoria, pp. 295–310.

Carboni, G. 2002, Ceramica: confronti per gruppi tipologici, in Manfredini, A. (ed), *Le dune, il lago, il mare: una comunità di villaggio dell’età del Rame a Maccarese*, Firenze 2002, pp. 144–165.

Cavalier, M. 1989, Catalogo dei materiali rinvenuti in stratigrafia, in Bernabò Brea, L. (ed), *La Grotta Cardini (Praia a Mare - Cosenza): giacimento del Bronzo,* Roma 1989*,* pp. 27–55.

Cocchi Genick, D. 1987, *Il riparo del Lauro di Candalla nel quadro del Bronzo medio iniziale dell’Italia centro-occidentale*, Viareggio 1987.

Cocchi Genick, D. 1992, La media età del Bronzo al Riparo Grande (Camaiore, Lucca), «Origini», XV, pp. 283–300.

Cocchi Genick, D. 1995, Rapporti tra la facies di Grotta Nuova e il Protoappenninico, in Cocchi Genick, D. (ed), *Aspetti culturali della media età del Bronzo in Italia centro-meridionale*, Firenze 1995, pp. 429–439.

Cocchi Genick, D. 1998, *L’antica età del Bronzo nell’Italia centrale. Profilo di un’epoca e di un’appropriata strategia metodologica*, Firenze 1998.

Cocchi Genick, D. 2001, *Classificazione tipologica e processi storici. Le ceramiche della facies di Grotta Nuova*, Viareggio 2001

Cocchi Genick, D. 2002, *Grotta Nuova : la prima unità culturale attorno all’Etruria protostorica*, Viareggio 2002.

Cremonesi, G. 2001, *La grotta sepolcrale eneolitica di San Giuseppe all’Isola d’Elba*, Pisa 2001.

Cuda, M.T., Sarti, L. 1996, Santa Maria in Belverde (Cetona): l’orizzonte dell’antica età del Bronzo, in Cocchi Genick, D. (ed), *L’antica età del Bronzo in Italia*. Firenze 1996, pp. 441–448.

Damiani, I. 1995, La facies protoappenninica, in Cocchi Genick, D. (ed), *Aspetti culturali della media età del Bronzo in Italia centro-meridionale*, Firenze 1995, pp. 398–428.

Esposito, E. 1990, La ceramica, in Albore Livadie, C. (ed), Archeologia a Piano di Sorrento. Ricerche di preistoria e protostoria nella penisola salentina, catalogo della mostra, Napoli 1990, pp. 53–81.

Feiken, H., Tol, G.W., Leusen, M. Van, Anastasia, C. 2012, Reconstructing a Bronze Age hidden landscape: geoarchaeological research at Tratturo Canio (Italy, 2009), «Palaeohistoria», 53/54, pp. 109–159.

Franco, M.C. 1982, *L’insediamento preistorico del Lago di Mezzano*. Roma 1982.

Guidi, A. 1980, Rinvenimenti preistorici nel territorio della Soprintendenza del Lazio, «Archeologia Laziale», X, pp. 38–42.

Guidi, A. 1981, Nuovi rinvenimenti in siti del passaggio alla media età del Bronzo, «Archeologia Laziale», IV, pp. 47–55.

Ingravallo, E., Piccinno, A. 1985, L’insediamento protoappenninico di Spigolizzi (Salve), «Studi di Antichità», 4, pp. 37–66.

Pennacchioni, M., Torre Crognola, in Pennacchioni M., D’Ercole V. 1977 (eds), *Vulci. Rinvenimenti di superficie d’epoca preistorica*, Roma, pp. 5–68.

Petitti, P., Mitchell, E. 1993, Dati preliminari sulla topografia dell’abitato sommerso del Lago di Mezzano, in A Baffetti A., Carancini G. L., Conti A. M. (eds), *Vulcano a Mezzano: insediamento e produzioni artigianali nella media Valle del Fiora nell’età del Bronzo*, Valentano 1993, pp. 17–31.

Prati, L. 1992, L’insediamento di Coriano (Forlì): la fase del Bronzo medio, «Rassegna di Archeologia», 10, pp. 670–671.

Soriano, E., Albore Livadie, C. 2017, La facies di Palma Campania e i suoi rapporti con le facies coeve dell’Italia medio-tirrenica e dell’Italia meridionale: considerazioni alla luce delle recenti scoperte, in Pontrandolfo A., Scafuro M. (eds), *Dialoghi sull’archeologia della Magna Grecia e del Mediterraneo* (Paestum 2016), pp. 101-111.

Talamo, P. 1996, Camposauro, in Cocchi Genick, D. 1996 (eds), *L’antica Età Del Bronzo in Italia*, Firenze 1996, pp. 576–577.
